# Supplementary material for: BASHY Dye Platform Enables the Fluorescence Bioimaging of Myelin Debris Phagocytosis by Microglia during Demyelination
Source: Cells. 2021 Nov 13;10(11):3163. doi: 10.3390/cells10113163 (PMC8620345; doi:10.3390/cells10113163)
Supplement: Supplementary file 1 [file cells-10-03163-s001.zip › cells-1400659-supplementary.pdf]

Supplementary Data

BASHY dye platform enables the fluorescence bioimaging of myelin debris phagocytosis by microglia during demyelination

Maria Vaz Pinto, Fábio M. F. Santos, Catarina Barros, Ana Rita Ribeiro, Uwe Pischel, Pedro M. P. Gois, Adelaide Fernandes

Contents of Supplementary Data include:

Supplementary Results (Supplementary Figures S1 to S5)

Supplementary Methods including structural characterization of BASHY td**1-4** (Supplementay Figures S6 to S39 and Supplementary Table S1)

Supplementary Results

List of supplementary Figures:

Supplementary Figure S1. Fluorescence of BASHY candidates synthesized for the detection of myelin debris.

Supplementary Figure S2. BASHY fluorescence decreases in control OCSC and mostly co-localizes with phagocytic Iba1+cells.

Supplementary Figure S3. LPC induces a shift in cell morphology towards a more amoeboid state and fosters an early pro-inflammatory activation of Iba1-positive cells.

Supplementary Figure S4. Evaluation of BASHY labeling in vivo.

Supplementary Figure S5. Evaluation of BASHY labeling in vivo, 23 days-post EAE immunization.

Supplementary Methods

List of Supplementary Figures:

Supplementary Figure S6. Preparation of ligand **1.**

Supplementary Figure S7. Preparation of BASHY td**1.**

Supplementary Figure S8. Preparation of salicylhydrazone **5**

Supplementary Figure S9. Preparation of BASHY td**2.**

Supplementary Figure S10. Preparation of phenylglyoxylic acid **6**.

Supplementary Figure S11. Preparation of BASHY td**3.**

Supplementary Figure S12. Preparation of phenylglyoxylic acid **7**.

Supplementary Figure S13. Preparation of BASHY td**4**

Supplementary Figure S14. ^{1}H-NMR of the compound **1′**.

Supplementary Figure S15. ^{13}C-NMR of the compound **1′**

Supplementary Figure S16. Low Resolution Mass Spectrum of the compound **1′**

Supplementary Figure S17. ^{1}H-NMR of BASHY td**1**

Supplementary Figure S18. ^{13}C-NMR of BASHY td**1**

Supplementary Figure S19. Low Resolution Mass Spectrum of BASHY td**1**.

Supplementary Figure S20. ^{1}H-NMR of compound **5′**

Supplementary Figure S21. ^{1}H-NMR of compound **5″**

Supplementary Figure S22. ^{13}C-NMR of compound **5″**

Supplementary Figure S23. Low Resolution Mass Spectrum of compound **5″**.

Supplementary Figure S24. ^{1}H-NMR of compound **5**

Supplementary Figure S25. ^{13}C-NMR of compound **5**

Supplementary Figure S26. High Resolution Mass Spectrum of compound **5**.

Supplementary Figure S27. ^{1}H-NMR of BASHY td**2**

Supplementary Figure S28. ^{13}C-NMR of BASHY td**2**

Supplementary Figure S29. High Resolution Mass Spectrum of BASHY td**2**

Supplementary Figure S30. ^{1}H-NMR of compound **6**

Supplementary Figure S31. Low Resolution Mass Spectrum of compound **6**.

Supplementary Figure S32. ^{1}H-NMR of BASHY td**3**

Supplementary Figure S33. ^{13}C-NMR of BASHY td**3**

Supplementary Figure S34. High Resolution Mass Spectrum of BASHY td**3**.

Supplementary Figure S35. ^{1}H-NMR of compound **7**

Supplementary Figure S36. Low Resolution Mass Spectrum of compound **7**.

Supplementary Figure S37. ^{1}H-NMR of BASHY td**4**

Supplementary Figure S38. ^{13}C-NMR of BASHY td**4**

Supplementary Figure S39. High Resolution Mass Spectrum of BASHY td**4**.

List of Supplementary Tables:

Supplementary Table S1. Selected photophysical data of the td**1-4** in apolar medium (toluene).

Supplementary Results

Supplementary Figure S1

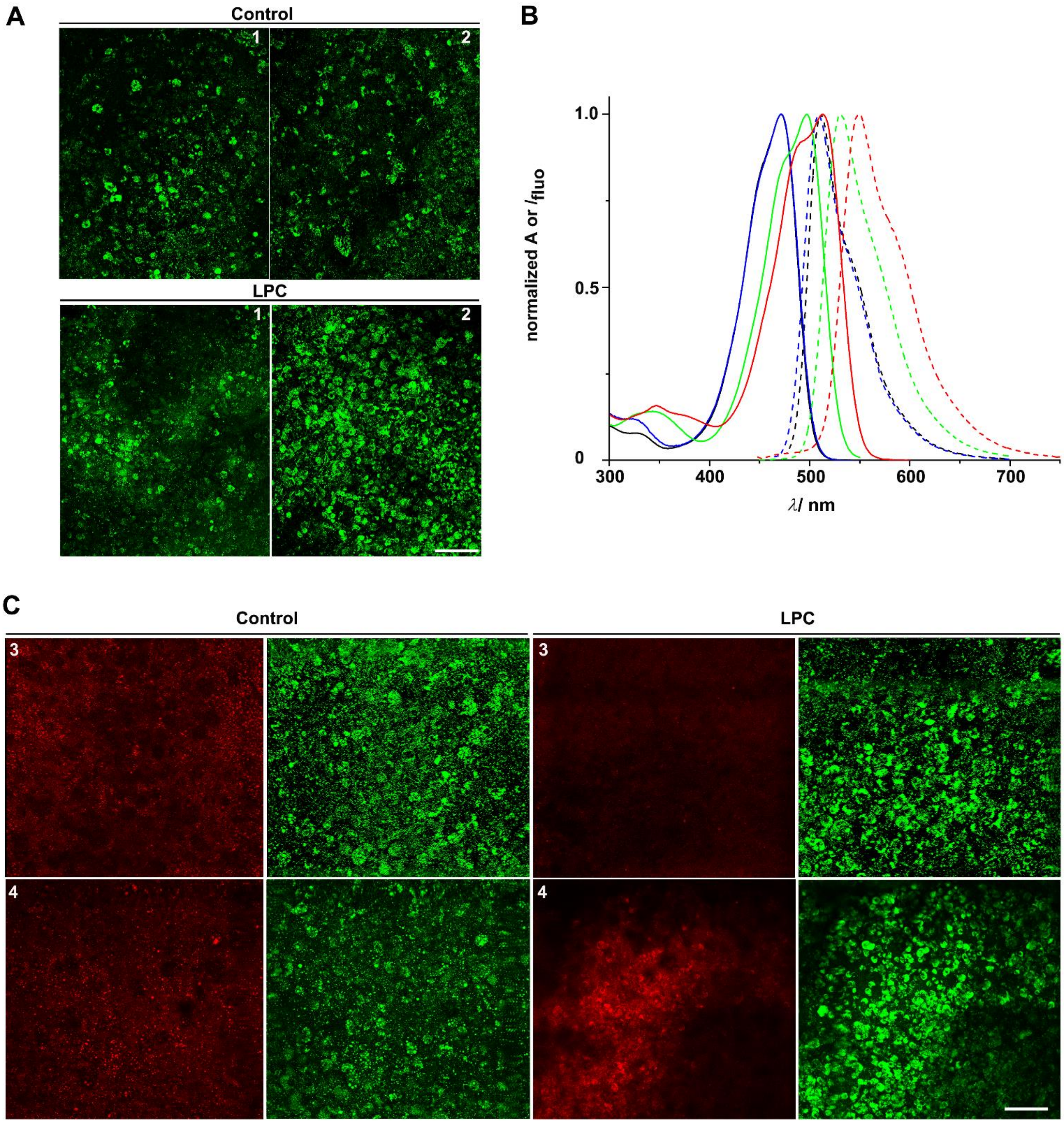

Supplementary Figure S1. Fluorescence of BASHY candidates synthesized for the detection of myelin debris. **A** Representative images of the different performances of BASHY td**1-2**, (green), in control and treated OCSC, 48 h after LPC induction. Scale bars equal 200 μm. **B** Normalized UV/vis absorption (solid lines) and fluorescence spectra (dashed lines) of the dyes td**1** (black), td**2** (blue), td**3** (green), and td**4** (red) in toluene solution. Please note that the spectra of td**1** and td**2** practically overlap. **C** Representative images of the different performances of BASHY td**3** and td**4** (red/green), in control and treated OCSC, 48 h after LPC induction, imaged in the cerebellar White Matter. Scale bars equal 200 μm.

Supplementary Figure S2

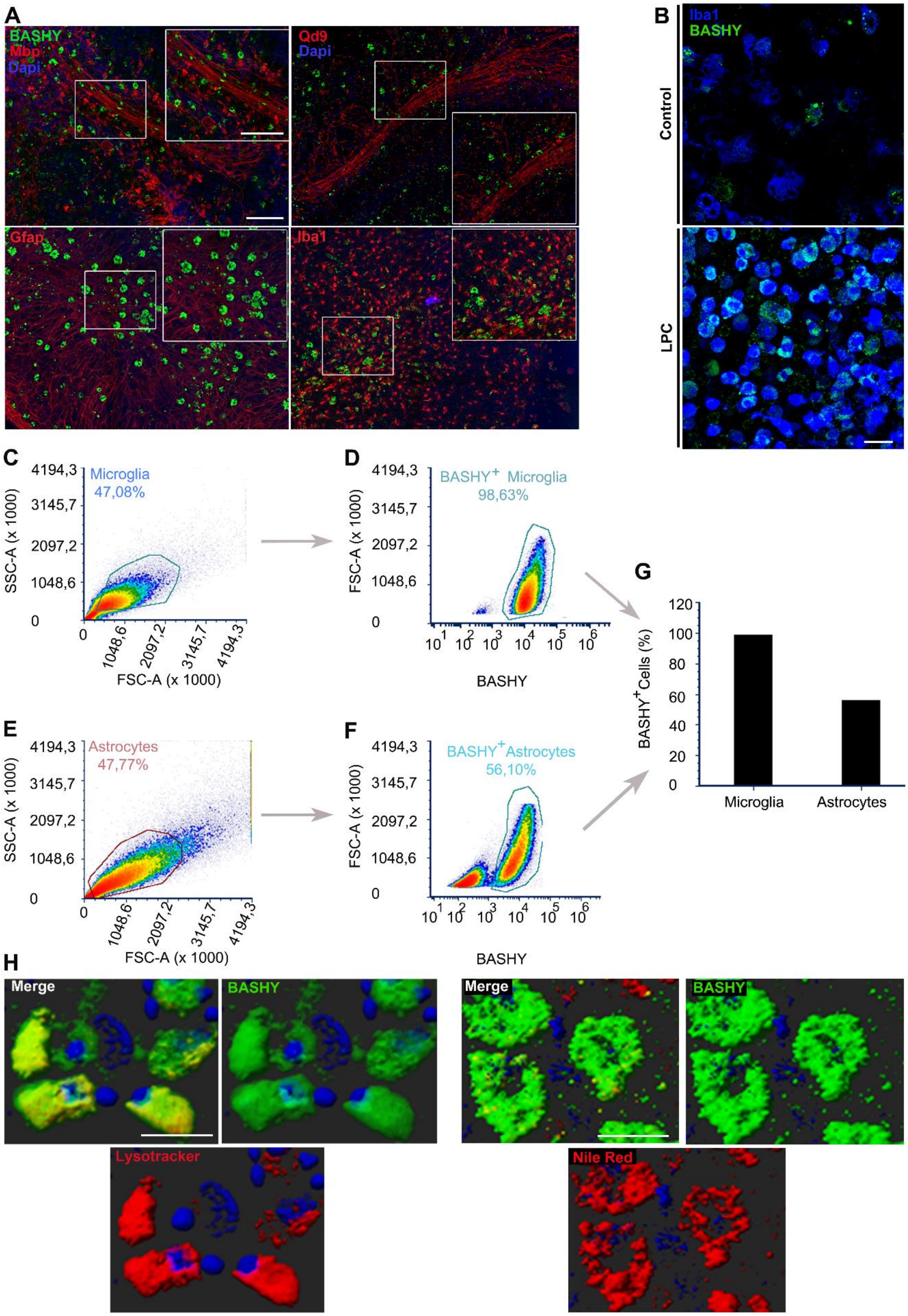

Supplementary Figure S2. BASHY fluorescence is reduced in control OCSC and mostly co-localizes with phagocytic Iba1⁺cells. A Representative images of BASHY (green) co-localization with mature oligodendrocytes/compact myelin (Mbp, red), degenerated myelin (Qd9, red), astrocytes (Gfap, red) and microglia/macrophages (Iba1, red), in non-treated OCSC at 48 h (magnification 20X). Slight co-localization of BASHY with Iba1-positive cells resulting from accumulation of myelin debris and early activation of macrophages/microglia as a result of the mechanical injury and cell-death associated with the cerebellar slicing procedure. Scale bar equals 120 μm. **B** Representative images of the co-localization of BASHY fluorescent signal with macrophage/microglial cells (Iba1, blue). Magnification 40x. Scale bar equal 20 μm. Gfap, glial fibrillar acidic protein; Mbp, myelin basic protein; Iba1, ionized calcium binding adaptor molecule 1. **C** Flow cytometry analysis comparing the capacity of human 10B1 astrocytes and human CHME3 microglia to phagocytose MD-BASHY. Gating strategy to detect Human CHME3 microglial cells. **D** Gating strategy to detect BASHY-positive Human CHME3 cells**. E** Gating strategy to detect Human 10B1 astrocytes. **F** Gating strategy to detect BASHY-positive Human 10B1 cells. **G** Quantitative analysis of MD-BASHY internalization by Astrocytes and microglia analyzed using FCS Express Flow Cytometry Software. **H** Representative 3D images from OCSC 48h-post LPC induction showing BASHY co-localization with Lysotracker and Nile Red. Visualized using Imaris software.

Supplementary Figure S3

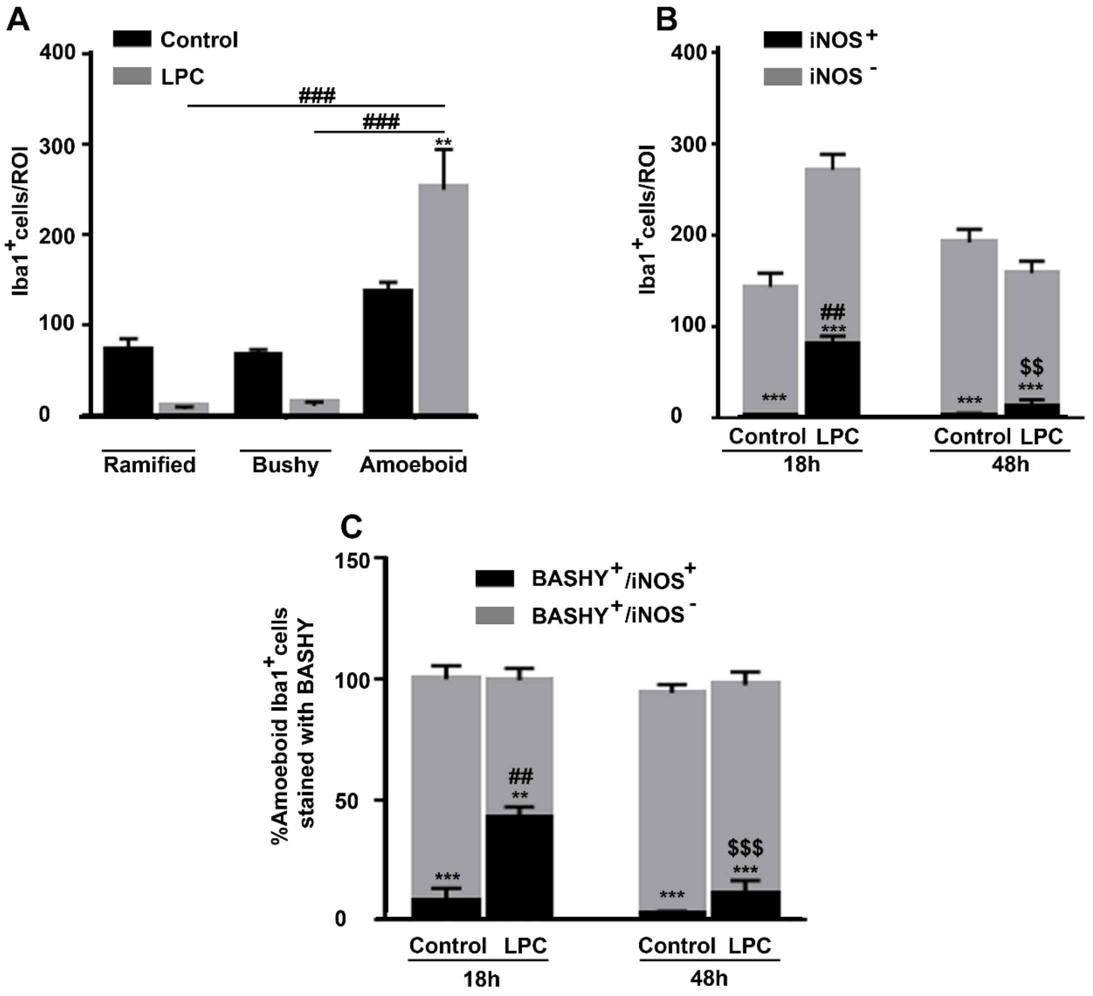

Supplementary Figure S3. LPC induces a shift in cell morphology towards a more amoeboid state and fosters an early pro-inflammatory activation of Iba1-positive cells. A Quantitative analysis of the differential phenotypes of macrophage/microglial Iba1-positive cells, including ramified, bushy and amoeboid. Results are mean ± SEM. **P<0.01 vs. respective Control; $^{\#\#\#}$P<0.01 for amoeboid LPC vs. Bushy LPC; and for amoeboid LPC vs. ramified LPC. **B** Quantitative analysis of the total number of Iba1-positive

macrophage/microglia per ROI with each phenotypes ($iNOS^+$, pro-inflammatory; $iNOS^-$, Anti-inflammatory) at both time points. Results are mean ± SEM. ***P<0.001 for $iNOS^+$ vs. respective $iNOS^-$; $^{\#\#}$P<0.01 for LPC vs. respective control; $^{\$\$}$P<0.01 for $iNOS^+$ LPC 48 h vs. $iNOS^+$ LPC 18 h. **C** Quantitative analysis of BASHY co-localization with anti-inflammatory ($iNOS^-$) and pro-inflammatory ($iNOS^+$) microglia at each time point. Results are mean ± SEM. **P<0.01 and ***P<0.001 for $BASHY^+/iNOS^+$ vs. respective $BASHY^+/iNOS^-$; $^{\#\#}$P<0.01 for $BASHY^+/iNOS^+$ LPC vs. respective Control; $^{\$\$\$}$P<0.001 for $BASHY^+/iNOS^+$ LPC 48 h vs. $BASHY^+/iNOS^+$ LPC 18 h.

Supplementary Figure S4

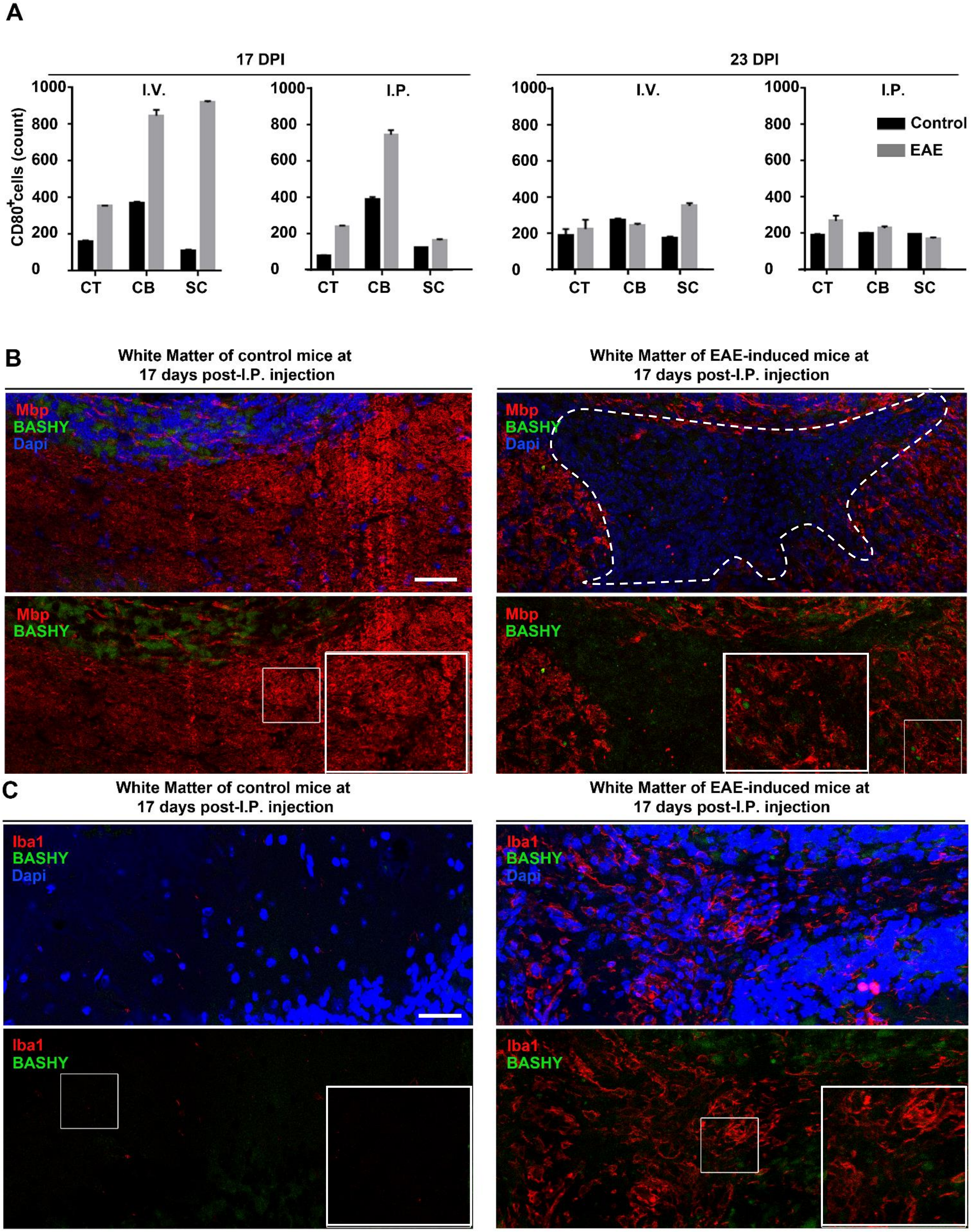

Supplementary Figure S4. Evaluation of BASHY labeling *in vivo*. A Flow cytometry analysis was performed using cells from Cortex (CT), Cerebellum (CB), and Spinal Cord (SC) from control and EAE-induced mice injected with BASHY. Cells were probed with the antibody CD80 to stain for activated microglia**. B** Representative images of brain sections from control and EAE-induced mice after 17 DPI, injected with BASHY I.P**.** Brain sections were stained for cell nuclei (DAPI, blue), and microglia/macrophages (Iba1, red) in, and imaged in the cerebellar White Matter to observe BASHY colocalization with lesion associated myelin-phagocytosing cells. Magnification 20X. Scale bar equals 200 μm.

Supplementary Figure S5

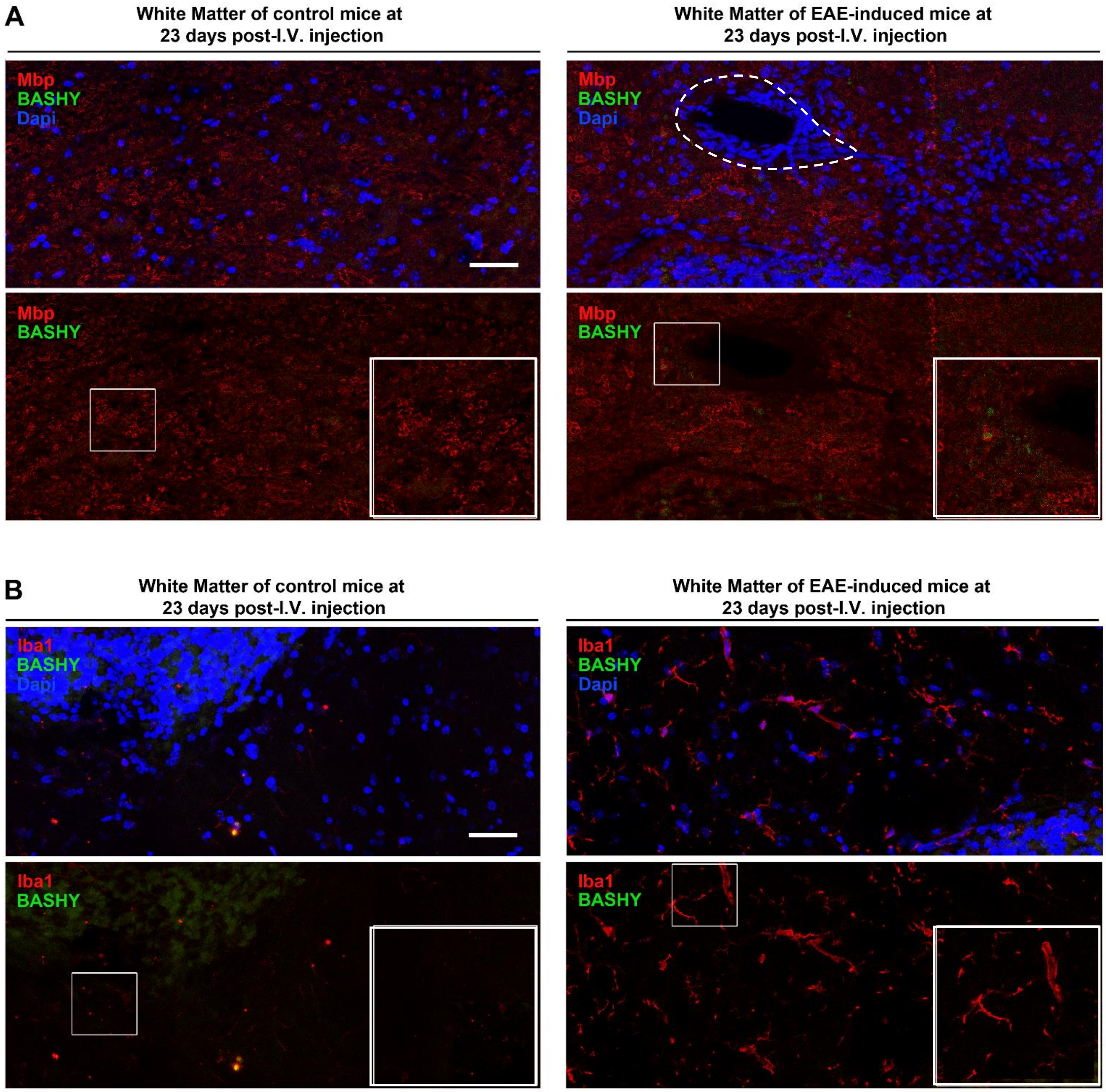

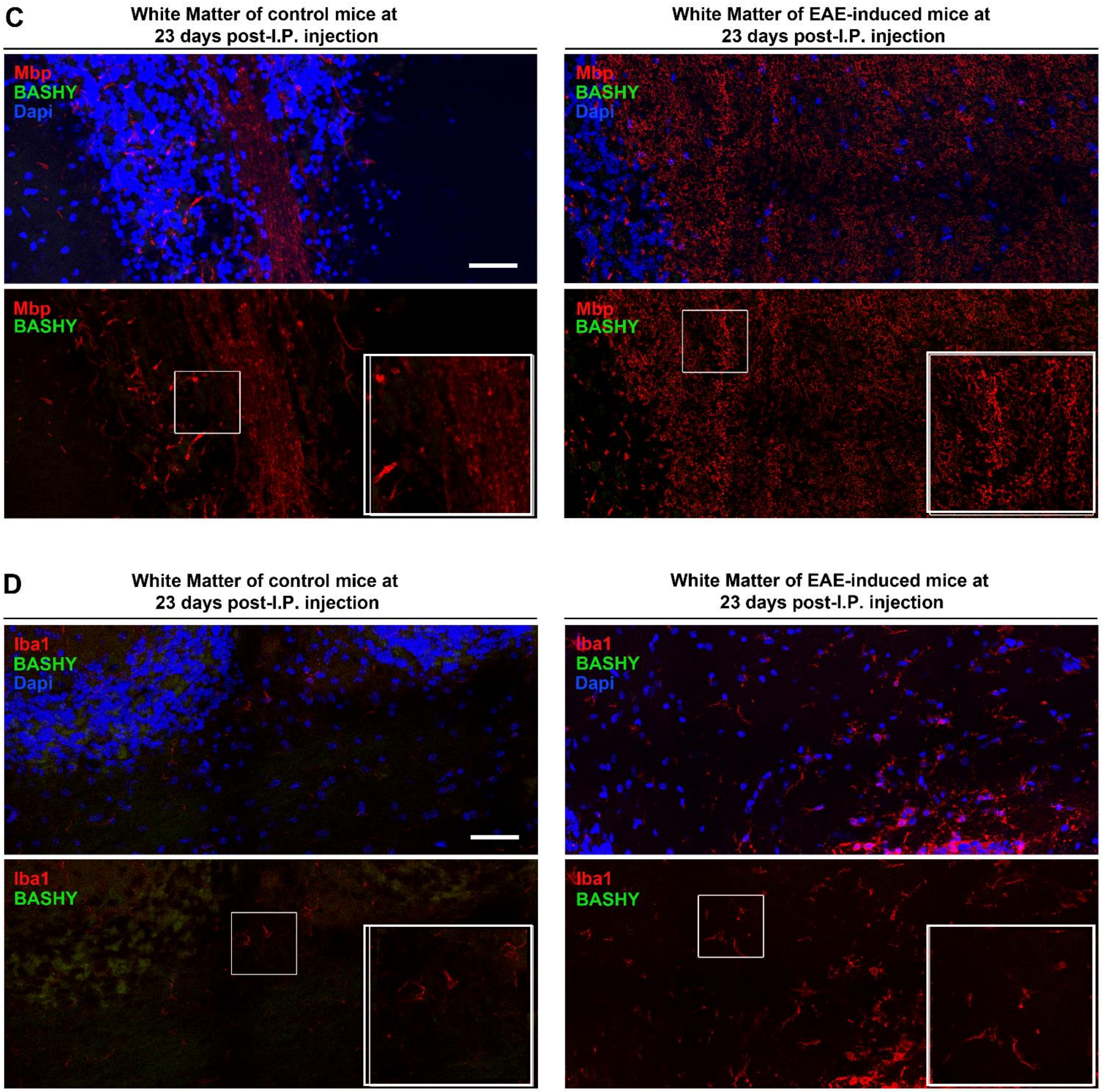

Supplementary Figure S5. Evaluation of BASHY labeling *in vivo,* 23 days-post EAE immunization. Representative images of brain sections from control and EAE-induced mice after 23 DPI, injected with BASHY I.V in **A** and **B** and I.P in **C** and **D**. Brain sections were stained for cell nuclei (DAPI, blue), compact myelin sheaths (myelin basic protein, Mbp, red) in **A** and **C,** or microglia/ macrophages (Iba1, red) in **B** and **D**, and imaged in the cerebellar White Matter to observe BASHY distribution towards demyelinated areas (areas with large accumulation of cells) and further colocalization with lesion associated myelin-phagocytosing cells. Magnification 20X. Scale bar equals 200 μm.

Supplementary Methods

BASHY synthesis and characterization

Material and methods:

The solvents acetonitrile, ethanol, and methanol were used without any purification. Dichloromethane was dried over calcium hydride under nitrogen atmosphere. Phenylboronic acid, hydrazine hydrate, 3-diethylaminophenol, propionic anhydride, 4-dimethylaminopyridine (DMAP), cinnamaldehyde, benzaldehyde and sodium pyruvate were purchased from Sigma-Aldrich. Phenylglyoxylic acid was purchased from Fluorochem. Aluminium trichloride was supplied by Alfa Aesar. Thin-layer chromatography was performed using Merck silica gel 60F254 aluminium plates, visualized using UV light and revealed with phosphomolybdic acid solution. For column chromatography silica gel 60 M was used, purchased from Macherey.

NMR spectra were recorded with a Bruker AMX 400 or a Bruker Fourier 300 using $CDCl_3$ or$(CD_3)_2SO$ as deuterated solvents. All coupling constants are expressed in Hz and chemical shifts (δ) in ppm. The multiplicity is given as: s (singlet), d (doublet), dd (double doublet), t (triplet), q (quartet) and m (multiplet). Low-resolution mass spectrometry (LRMS) was performed with an ion-trap mass analyzer (Thermo Scientific LCQ Fleet Ion Trap LC/MS) equipped with an electrospray interface and in a mass spectrometer (Micromass Quattro Micro API, Waters, Ireland) with a Triple Quadrupole (TQ) and with an electrospray ion source (ESI) operating in positive mode. High-resolution mass spectrometry (HRMS) was done with an Orbitrap Elite spectrometer (Thermo Scientific, UK), using ESI in positive mode. Elemental analysis (CHN) was performed with a Flash 2000 CHNS-O analyzer (Thermo Scientific, UK). Melting points were determined using a melting point apparatus (Stuart, SMP10).

.

Synthesis of Shiff base ligand 1″:

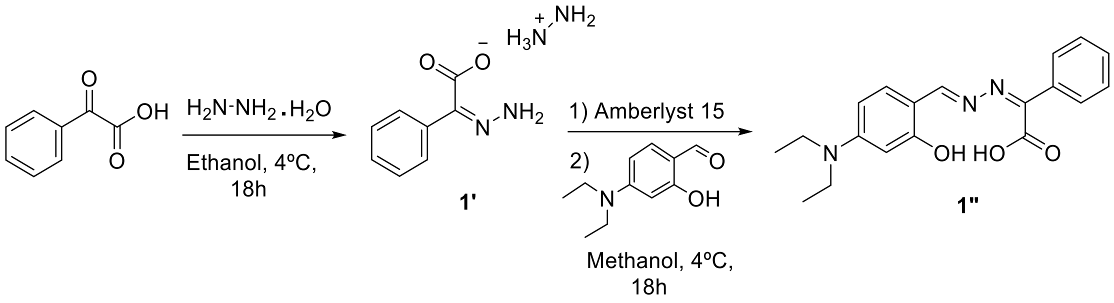

Supplementary Figure S6. Synthesis of ligand **1″**. Prepared as previously described [19]. In order to prepare hydrazonophenylacetate **1′**, hydrazine monohydrate (1.45 mL; 30 mmol) and phenylglyoxylic acid (1.5 g; 10 mmol) were each dissolved in a separate portion of ethanol (5 ml). Then, the two solutions were mixed into a round bottom flask and the reaction continued for 18 h at 4°C. The reaction mixture was then filtered and the crystaline white needles were washed with 2 mL of cold ethanol. Compound **1′** was obtained in 70% yield and was immediately used in the construction of the Schiff base ligand **1″**. Compound **1′** (0.5 g; 2.5 mmol) was dissolved in 5 mL of water and then 45 mL of methanol were added. This solution was slowly passed through a column packed with amberlyst 15 (3 ml of dry resin wetted with methanol) into a round bottom flask containing a 5 mL methanolic solution of 4-diethylaminosalicylaldehyde (0.483 g; 2.5 mmol). The resulting mixture was stirred for 2 h at room temperature and then it was left at 4°C for 18 h. Afterwards, the reaction mixture was filtered and the orange solid obtained was washed with 2 mL of cold methanol and with 2 mL of cold dichloromethane.

Synthesis of BASHY td1 (Method A):

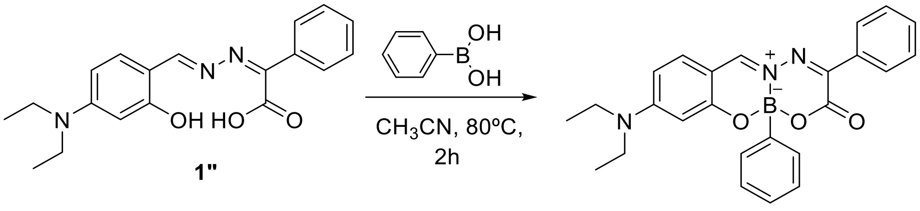

1" 1

Supplementary Figure S7. Synthesis of BASHY td**1**. Prepared as previously described in [19].

In order to assemble BASHY **1**, equimolar amounts of ligand **1"** (34 mg; 0.1 mmol) and phenylboronic acid (12 mg; 0.1 mmol) were added to a round-bottomed flask, dissolved in 1 mL of acetonitrile. The reaction mixture was stirred at 80ºC for 2 h and then the volatiles were evaporated.

Synthesis of salicylhydrazone 5:

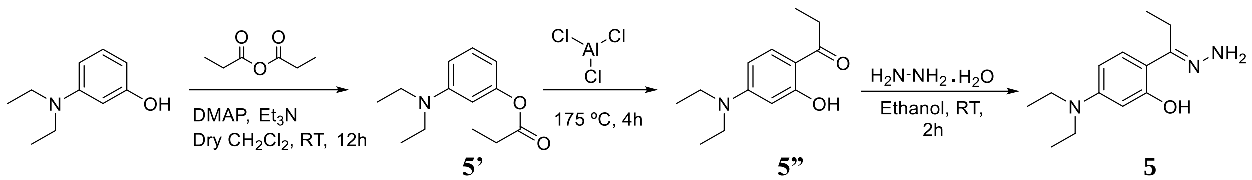

Supplementary Figure S8. Synthesis of salicylhydrazone **5**. Prepared as previously described in [30].

In the first step, a flame dried round-bottomed flask was loaded with 3-(diethylamino)phenol (0.85 g; 5.16 mmol) followed by 10 mL of dry dichloromethane. Then, propionic anhydride (1 mL; 8.25 mmol), *N,N*-dimethylpyridin-4-amine (0.032 g; 0.26 mmol), and triethylamine (0.86 mL; 6.19 mmol) were successively added and the reaction mixture was left to stir at room temperature for 12 h. Afterwards dichloromethane (100 mL) was added to the reaction, the mixture was washed with a saturated $NaHCO_3$ solution (2 × 30 mL), brine (2 × 30 mL), and dried with anhydrous $MgSO_4$. After filtration dichloromethane was removed under reduced pressure and the crude was purified by silica gel chromatography (hexane/ethyl acetate = 90/10).

Then, in a round-bottomed flask, **5'** (0.711 g; 3.22 mmol) and aluminum trichloride (1.3 g; 9.65 mmol) were mixed and heated at 175°C for 4 h. After cooling, the reaction mixture was treated with 14 mL of cold HCl solution (1 M). This mixture was stirred at 100ºC for 1 h and then 50 mL of ethyl acetate were added. The organic phase was collected and the remaining aqueous phase was extracted twice with 25 mL of ethyl acetate. The combined organic phases were dried over anhydrous Na_2SO_4. After filtration the volatiles were removed under reduced pressure. The crude was purified by silica gel column chromatography (hexane/ethyl acetate = 95/5).

In the final step, a round-bottomed flask was loaded with hydrazine hydrate (0.73 mL; 8.29 mmol) in 1 mL of ethanol and then a solution of **5"** (0.057 g; 0.258 mmol) in 1 mL of ethanol was added dropwise into this

solution. The reaction mixture was stirred for 1.5 h at room temperature and then the volatiles were evaporated under reduced pressure.

Synthesis of BASHY td2 (Method B):

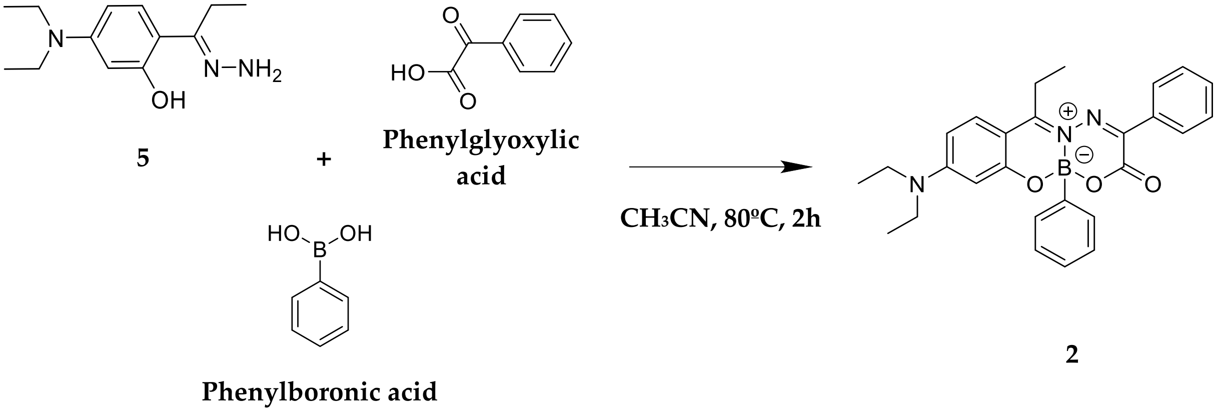

Supplementary Figure S9. Synthesis of BASHY td**2**. Prepared as previously described in [30].

To a round-bottomed flask it was dissolved in 1 mL of acetonitrile, equimolar amounts of **5** (0.024 g; 0.1 mmol), phenylglyoxylic acid (0.015 g; 0.1 mmol) and phenylboronic acid (0.012 g; 0.1 mmol). The reaction mixture was stirred at 80ºC for 2 h. Afterwards, volatiles were evaporated under reduced pressure and the crude mixture was re-dissolved in 1 mL of dichloromethane and purified by preparative thin layer chromatography, using dichloromethane as eluent.

Synthesis of phenylglyoxylic acid 6:

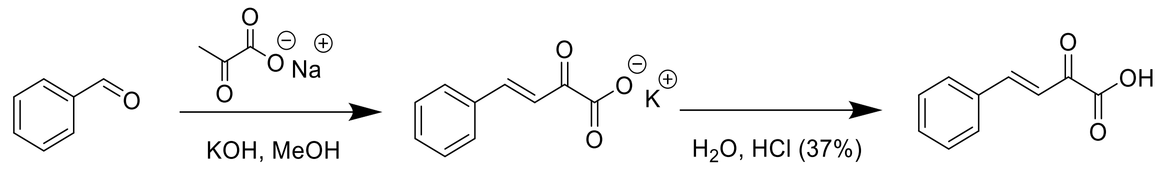

benzaldehyde **6′** **6**

Supplementary Figure S10. Synthesis of phenylglyoxylic acid **6**. Prepared as previously described in [30].

In a round-bottomed flask, sodium 2-oxopropanoate (1 g; 9.09 mmol) and benzaldehyde (0.93 mL; 9.09 mmol) were mixed in 3 mL of methanol at 0℃. Then 2.5 mL of a methanolic KOH solution (4 M) were slowly added, keeping the temperature below 5℃. After removing the ice bath another 1.5 mL of the KOH solution (4 M) was added and the reaction mixture was heated to 40°C for 45 min. The initial white precipitate turned yellow, which corresponds to the potassium salt **6′**. After filtration the solid was dried under reduced pressure. Compound **6′** (1.2 g; 5.52 mmol) was then dissolved in 20 mL of water at 0℃ and while stirring intensively, hydrochloric acid (37%, 2 mL) was added. Compound **6** precipitated immediately as a yellow solid, which was filtrated, washed with 5 mL of water and dried under reduced pressure.

Synthesis of BASHY td3 (Method B):

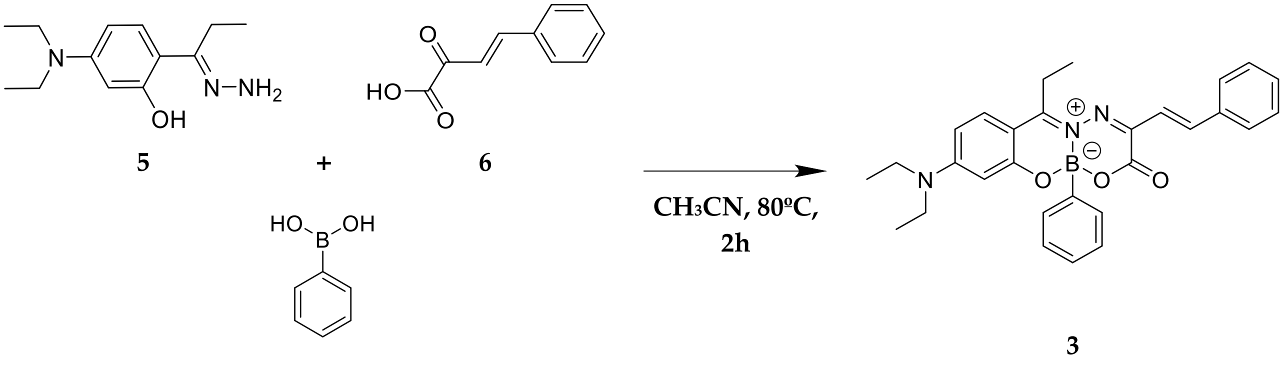

Phenylboronic acid

Supplementary Figure S11. Synthesis of BASHY td**3**. Prepared as previously described in [30].

To a round-bottomed flask it was dissolved in 1 mL of acetonitrile, equimolar amounts of **5** (0.024 g; 0.1 mmol), **6** (0.018 g; 0.1 mmol) and phenylboronic acid (0.012 g; 0.1 mmol). The reaction mixture was stirred at 80ºC for 2 h. Afterwards, volatiles were evaporated and reduced pressure and the crude mixture was re-dissolved in 1 mL of dichloromethane and purified by preparative thin layer chromatography, using dichloromethane as eluent.

Synthesis of phenylglyoxylic acid 7:

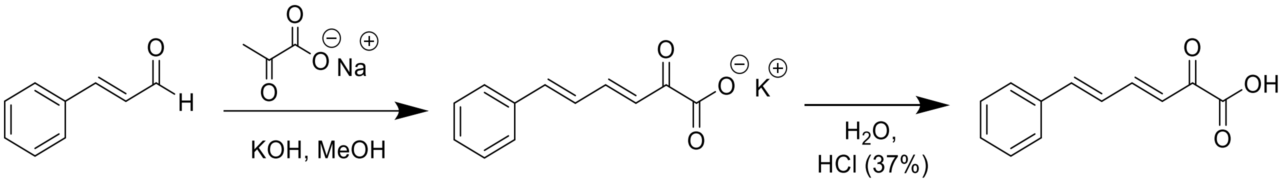

cinnamaldehyde **7′** **7**

Supplementary Figure S12. Synthesis of phenylglyoxylic acid **7**. Prepared as previously described in [30].

In a round-bottomed flask, sodium 2-oxopropanoate (1 g; 9.09 mmol) and cinnamaldehyde (1.14 mL; 9.09 mmol) were mixed in 3 mL of methanol at 0 ℃. Then 2.5 mL of a methanolic KOH solution (4 M) were slowly added, keeping the temperature below 5 ℃. After removing the ice bath another 1.5 mL of the KOH solution (4 M) were added and the reaction mixture was heated to 40°C for 45 min. The initial white precipitate turned yellow, which corresponds to the potassium salt **7′**. After filtration the solid was dried under reduced pressure. Compound **7′** (1.32 g; 5.52 mmol) was then dissolved in 20 mL of water at 0 ℃ and while stirring intensively, hydrochloric acid (37%, 2 mL) was added. Compound **7** precipitated immediately as an orange solid, which was filtrated, washed with 5 mL of water and dried under reduced pressure.

Synthesis of BASHY td4 (Method B):

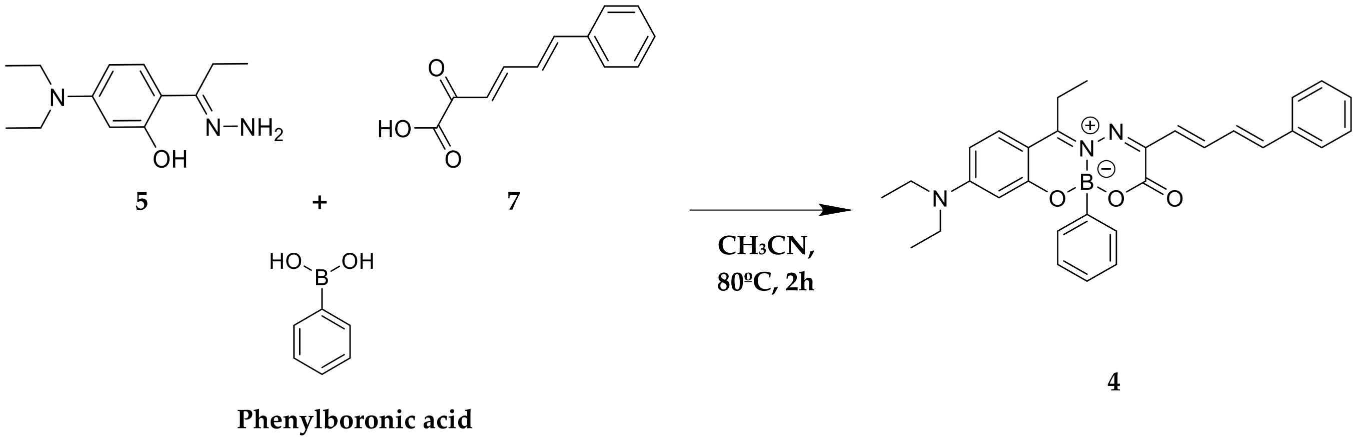

Supplementary Figure S13. Synthesis of BASHY td**4**. Prepared as previously described in [30].

To a round-bottomed flask it was dissolved in 1 mL of acetonitrile, equimolar amounts of **5** (0.024 g; 0.1 mmol), **7** (0.020 g; 0.1 mmol) and phenylboronic acid (0.012 g; 0.1 mmol). The reaction mixture was stirred at 80 ºC for 2 h. Afterwards, volatiles were evaporated under reduced pressure and the crude mixture was re-dissolved in 1 mL of dichloromethane and purified by preparative thin layer chromatography, using dichloromethane as eluent.

Structural characterization data

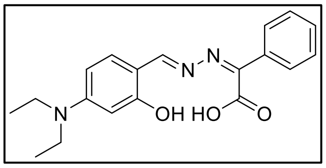

Ligand **1''** – Orange solid; Yield 60 %; ^{1}**H NMR** (300 MHz, $CDCl_3$) δ 8.14 (s, 1H, CH$_{imine}$), 8.05 – 8.00 (m, 2H, CH$_{Aromatic}$), 7.69 (d, *J* = 2.1 Hz, 1H, CH$_{Aromatic}$), 7.42 – 7.36 (m, 3H, CH$_{Aromatic}$), 7.17 (d, *J* = 9.3 Hz, 1H, CH$_{Aromatic}$), 6.33 (dd, *J* = 9.3, 2.1 Hz, 1H, CH$_{Aromatic}$), 3.56 (q, *J* = 6.9 Hz, 4H, CH_2's), 1.29 (t, *J* = 6.9 Hz, 6H, CH_3's); ^{13}**C NMR** (75 MHz, $(CD_3)_2SO$) δ 166.84, 164.47, 161.37, 159.07, 151.94, 134.28, 131.50, 131.34, 129.26, 129.18, 127.51, 126.87, 106.08, 104.52, 96.86, 44.03, 12.56; **LRMS** calcd m/z ($[M+H]^+$): 340, found m/z ($[M+H]^+$): 340; **Elemental analysis** calcd (%) for $C_{19}H_{21}N_3O_3.\frac{3}{5}H_2O$: C 65.16, H 6.39, N 12.00, found (%): C 65.54, H 6.08, N 11.61.

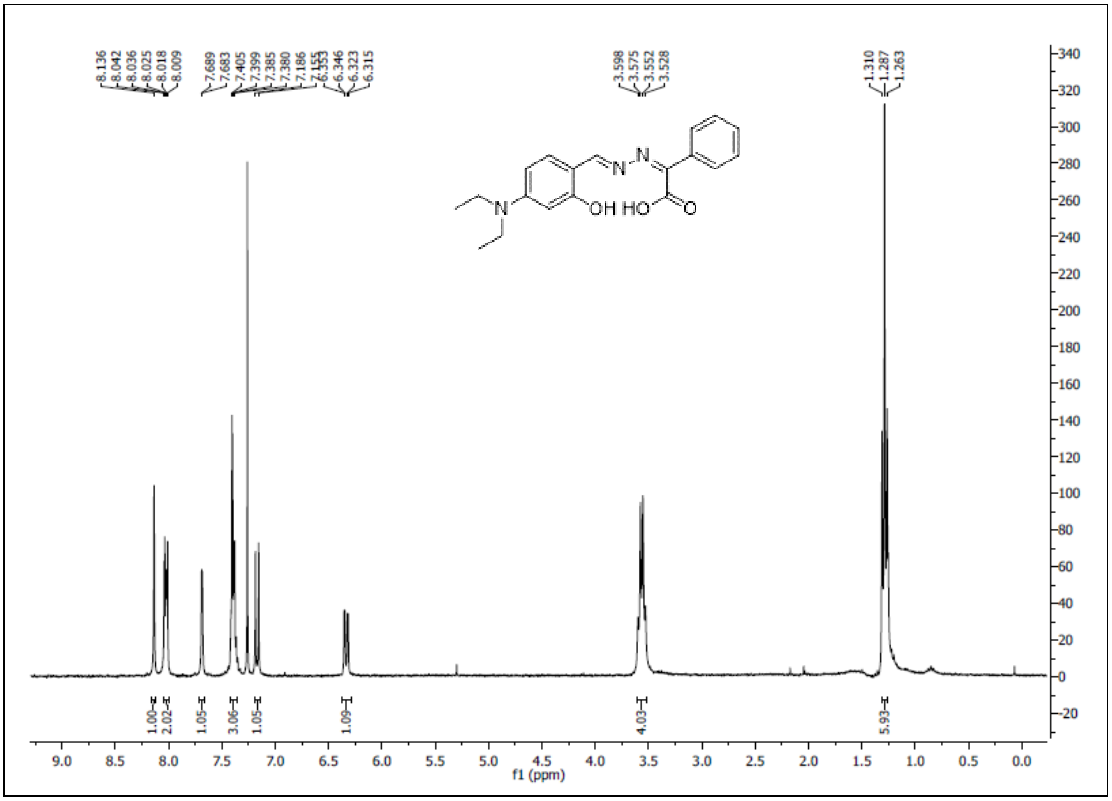

Supplementary Figure S14. ^{1}H-NMR of the compound **1'** (300 MHz, $CDCl_3$).

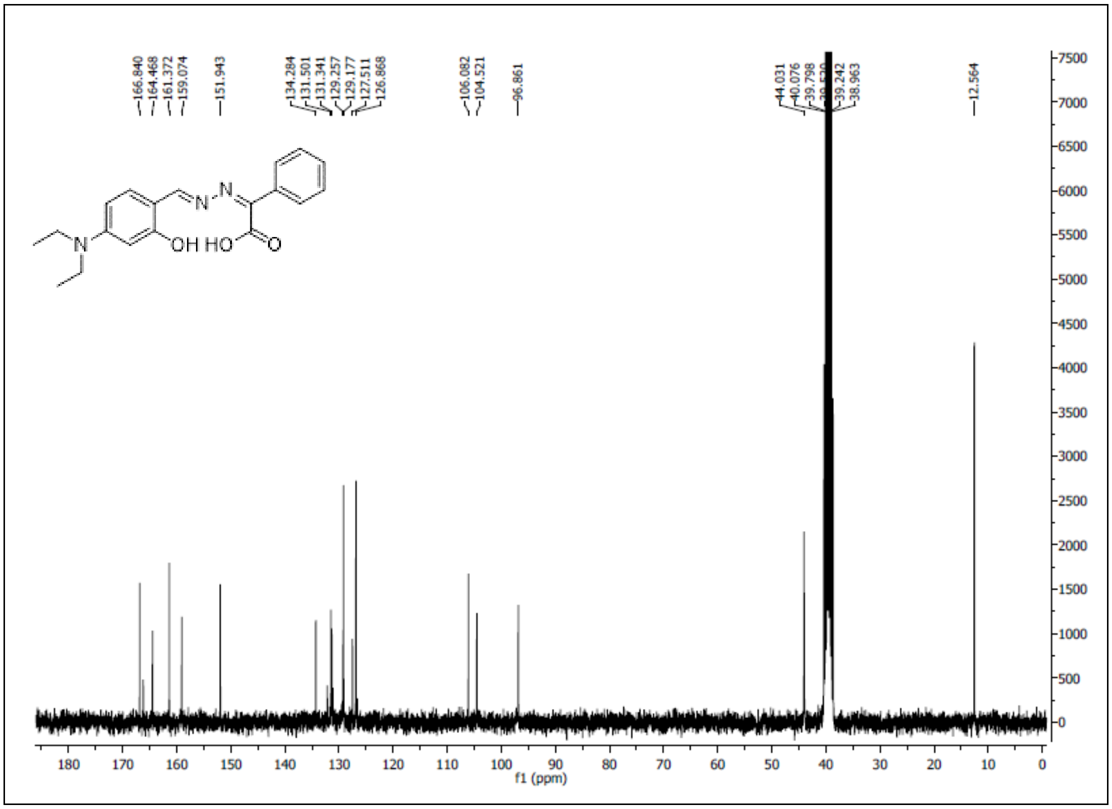

Supplementary Figure S15. ^{13}C-NMR of the compound **1′** (75 MHz, $(CD_3)_2SO$).

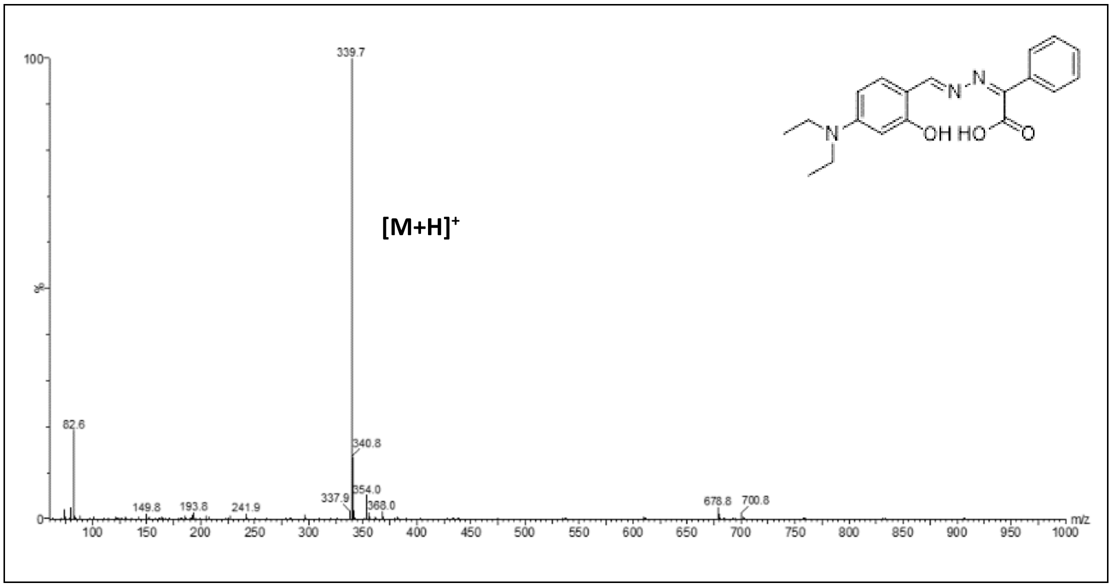

Supplementary Figure S16. Low Resolution Mass Spectrum of the compound **1′**.

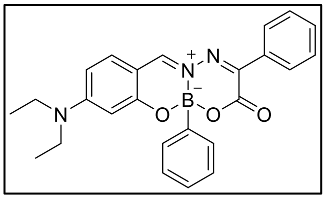

BASHY **1** – Orange solid; Yield 99 %; **^{1}H NMR** (300 MHz, $CDCl_3$) δ 8.29 (s, 1H, CH_{imine}), 8.00 (m, 2H, $CH_{aromatic}$), 7.45 – 7.35 (m, 5H, $CH_{aromatic}$), 7.24 – 7.18 (m, 4H, $CH_{aromatic}$), 6.37 (dd, *J* = 9.0, 2.4 Hz, 1H, $CH_{aromatic}$), 6.22 (d, *J* = 2.4 Hz, 1H, $CH_{aromatic}$), 3.52 – 3.39 (m, 4H, CH_2's), 1.24 (t, *J* = 7.2 Hz, 6H, CH_3's); **^{13}C NMR** (75 MHz, $CDCl_3$) δ 161.67, 157.38, 155.90, 154.03, 153.68, 134.70, 132.77, 130.93, 130.85, 129.56, 128.29, 127.92, 127.70, 107.80, 106.49, 98.74, 45.46, 12.83; **LRMS** calcd m/z ($[M+H]^+$): 426, found m/z ($[M+H]^+$): 426; **Elemental analysis** calcd (%) for $C_{25}H_{24}BN_3O_3$: C 70.6, H 5.69, N 9.88, found (%): C 70.8, H 5.98, N 9.89.

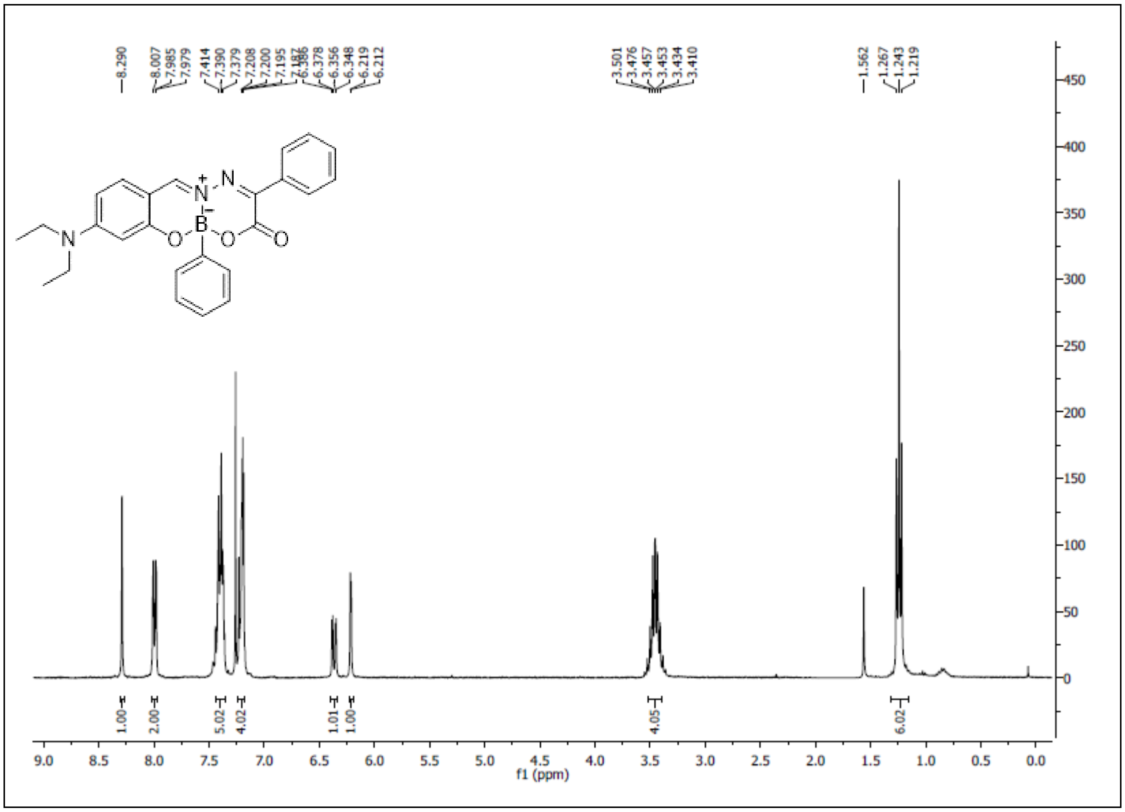

Supplementary Figure S17. ^{1}H-NMR of BASHY td**1** (300 MHz, $CDCl_3$).

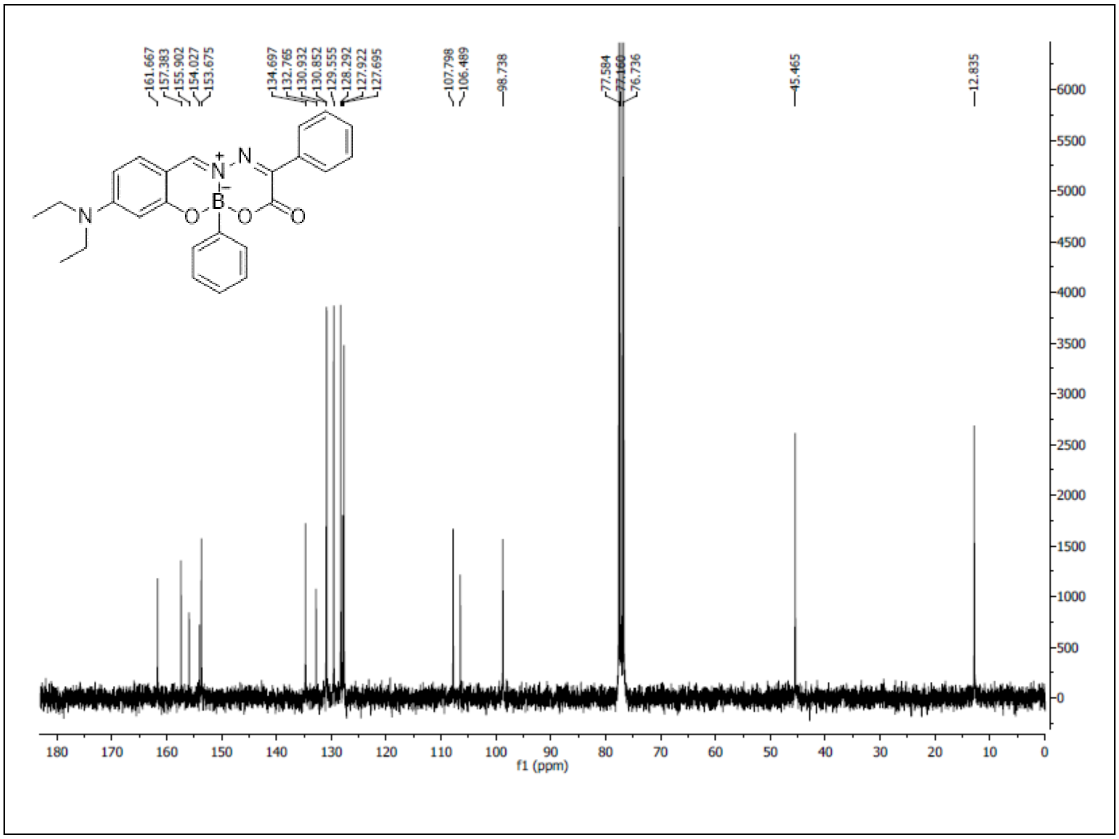

Supplementary Figure S18. ^{13}C-NMR of BASHY td**1** (75 MHz, $CDCl_3$).

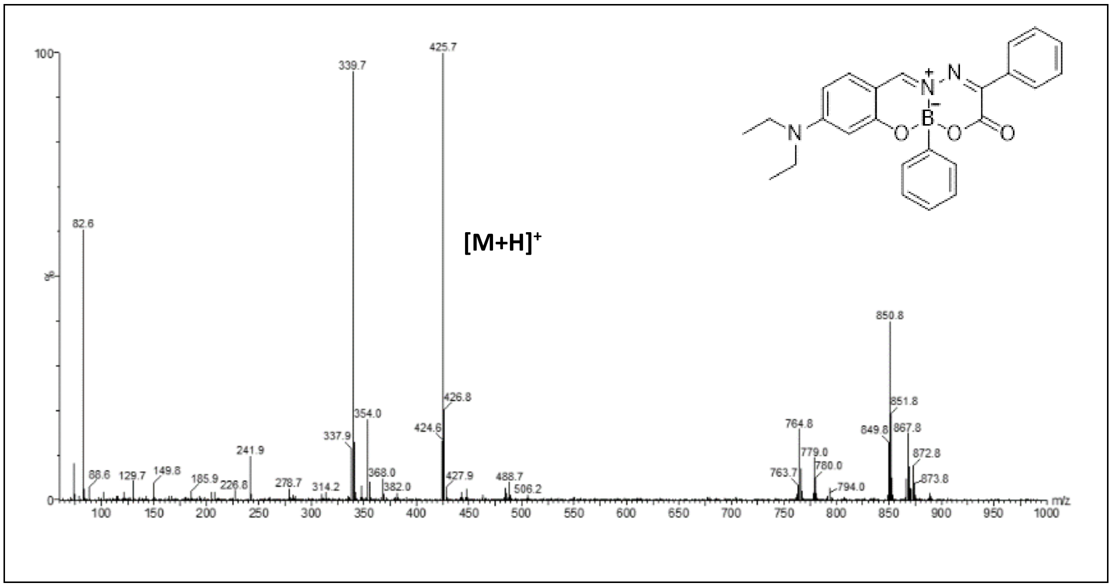

Supplementary Figure S19. Low Resolution Mass Spectrum of BASHY td**1**.

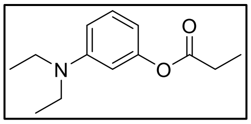

Compound **5'** – Colorless oil; Yield 62 %; [1]**H NMR** (300 MHz, $CDCl_3$) δ 7.16 (t, *J* = 9 Hz, 1H, $CH_{aromatic}$), 6.55 – 6.47 (m, 1H, $CH_{aromatic}$), 6.37 – 6.30 (m, 2H, $CH_{aromatic}$), 3.32 (q, *J* = 7 Hz, 4H, -N(C$\underline{H}_2$)$_2$(CH_3)$_3$), 2.57 (q, *J* = 8 Hz, 2H, -C C$\underline{H}_2$$CH_3$), 1.26 (t, *J* = 8 Hz, 3H, -CCH_2C$\underline{H}_3$), 1.15 (t, *J* = 7 Hz, 6H, -N(CH_2)$_2$(C$\underline{H}_3$)$_3$).

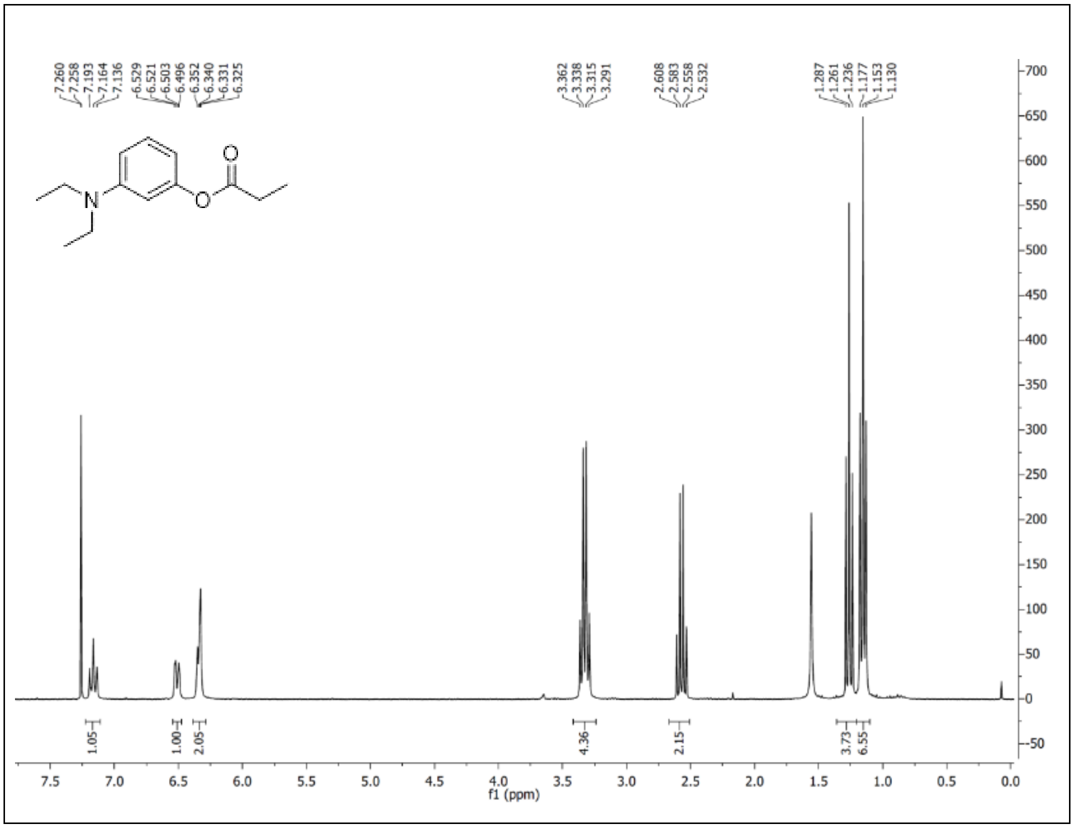

Supplementary Figure S20. ^{1}H-NMR of compound **5'** (300 MHz, $CDCl_3$).

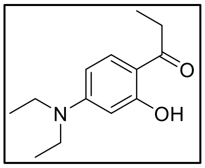

Compound **5″ –** Orange oil; Yield 32 %; **^{1}H NMR** (300 MHz, $CDCl_3$) δ 13.01 (s, 1H, -OH), 7.54 (d, *J* = 9 Hz, 1H, $CH_{aromatic}$), 6.18 (dd, *J* = 9, 3 Hz, 1H, $CH_{aromatic}$), 6.08 (d, *J* = 3 Hz, 1H, $CH_{aromatic}$), 3.39 (q, *J* = 7 Hz, 4H, -N(C$\underline{H}_2$)$_2$(CH_3)$_3$), 2.87 (q, *J* = 8 Hz, 2H, -C$\underline{H}_2$$CH_3$), 1.27 – 1.12 (m, 9H, -$CH_2C\underline{H}_3$ and -N(CH_2)$_2$(C$\underline{H}_3$)$_3$); **^{13}C NMR** (75 MHz, $CDCl_3$) δ 203.6, 165.3, 153.7, 131.9, 109.2, 103.6, 97.3, 44.7, 30.6, 12.7, 9.3; **LRMS** calcd m/z ($[M+H]^+$): 222, found m/z ($[M+H]^+$): 222.

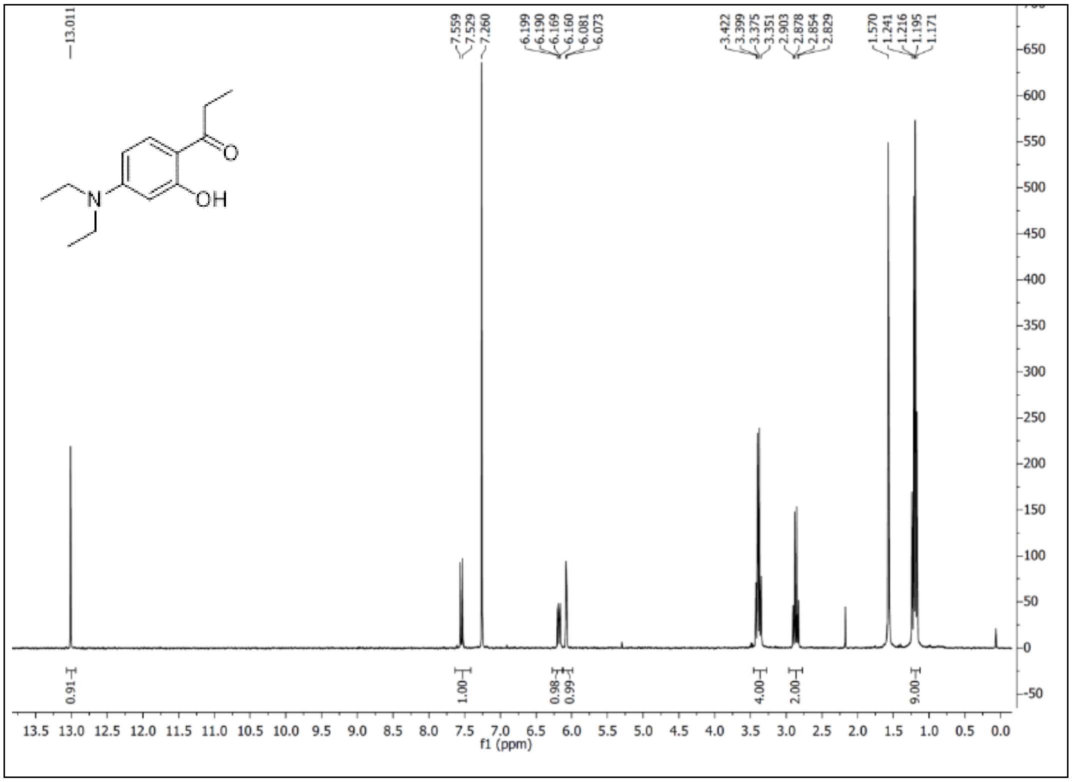

Supplementary Figure S21. ^{1}H-NMR of compound **5″** (300 MHz, $CDCl_3$).

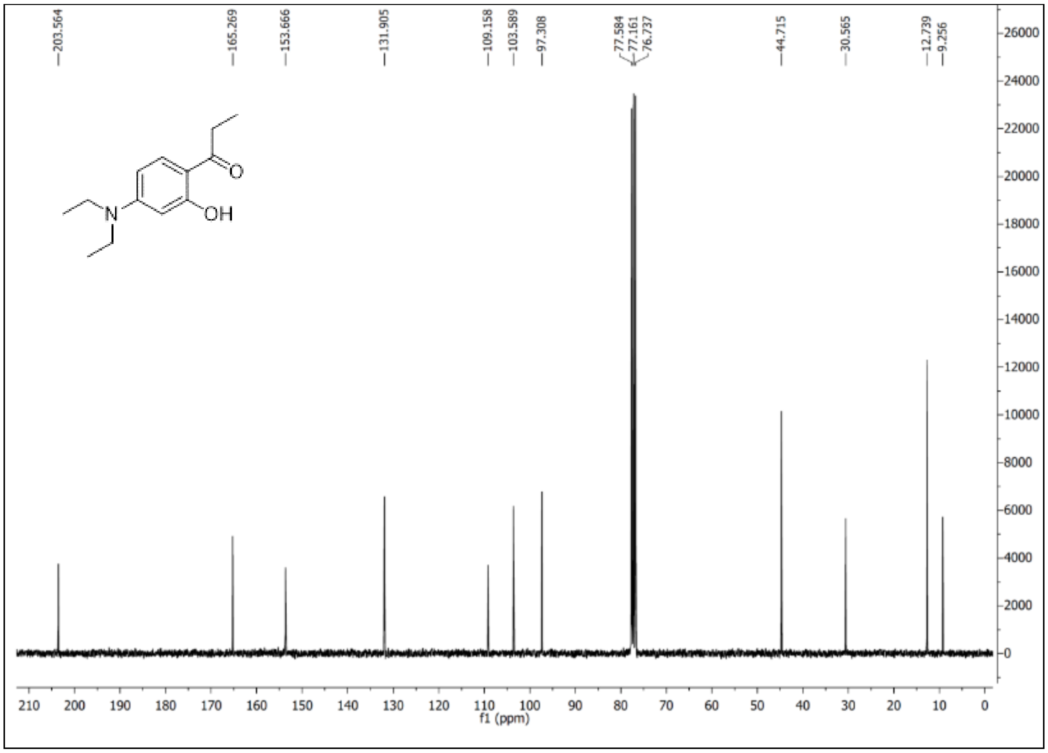

Supplementary Figure S22. ^{13}C-NMR of compound **5″** (75 MHz, $CDCl_3$).

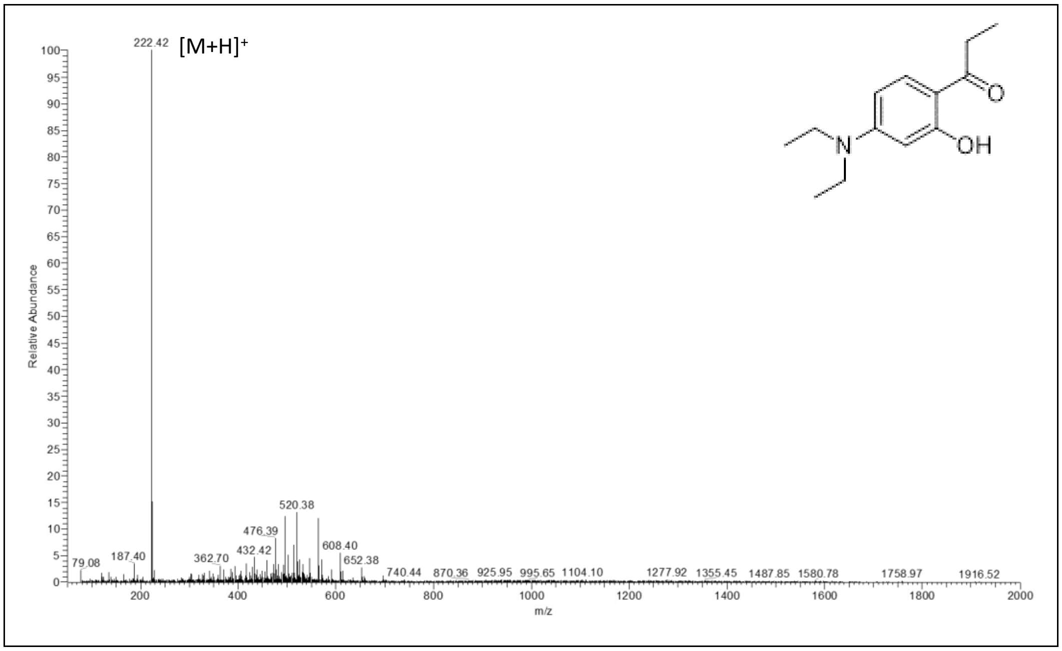

Supplementary Figure S23. Low Resolution Mass Spectrum of compound **5″**.

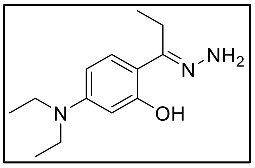

Compound **5.** Brown solid; Yield 99 %; **m.p.** 73-74 ºC; **^{1}H NMR** (300 MHz, $CDCl_3$) δ 13.14 (s, 1H, -OH), 7.19 (d, *J* = 8.7 Hz, 1H, $CH_{aromatic}$), 6.30 – 6.06 (m, 2H, $CH_{aromatic}$), 5.04 (s, 2H, $-NH_2$), 3.34 (q, *J* = 7.2 Hz, 4H, $-N(C\underline{H}_2)_2(CH_3)_3$), 2.66 (q, *J* = 7.8 Hz, 2H, $-CC\underline{H}_2CH_3$), 1.30 – 1.02 (m, 9H, $-CCH_2C\underline{H}_3$ and $-N(CH_2)_2(C\underline{H}_3)_3$); **^{13}C NMR** (75 MHz, $CDCl_3$) δ 161.02, 158.97, 149.30, 127.69, 107.59, 102.62, 99.17, 44.37, 17.68, 12.81, 10.44; **HRMS** calcd m/z ($[M+H]^+$): 236.1763, found m/z ($[M+H]^+$): 236.1741.

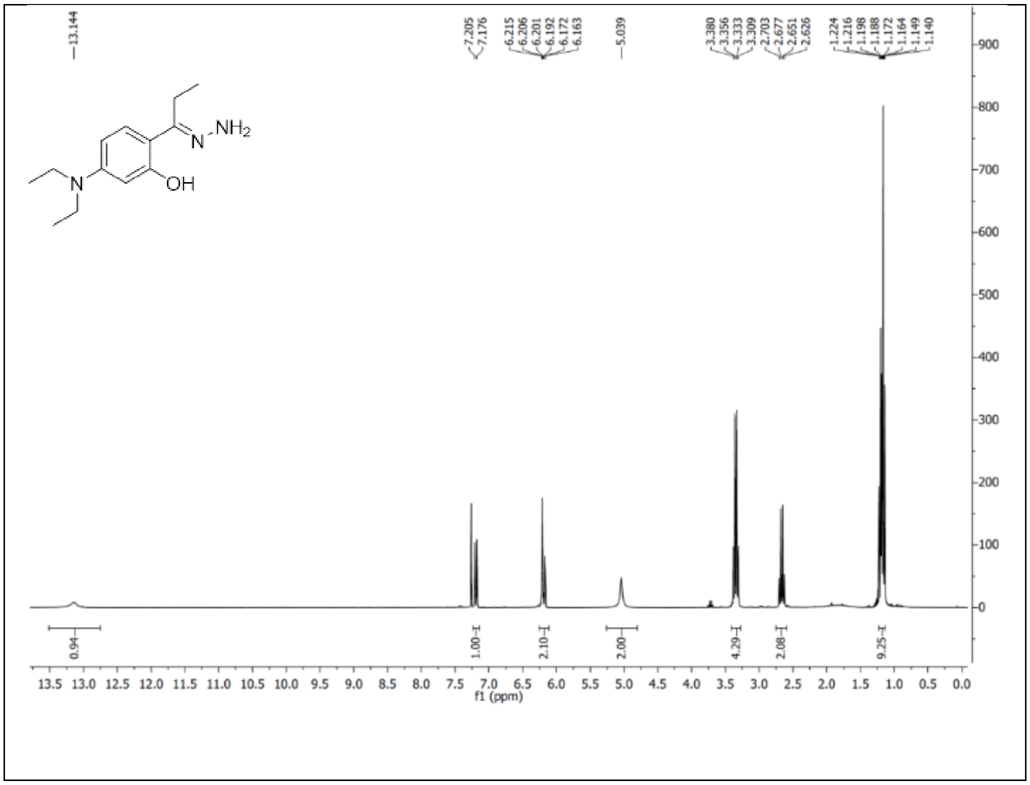

Supplementary Figure S24. ^{1}H-NMR of compound **5** (300 MHz, $CDCl_3$).

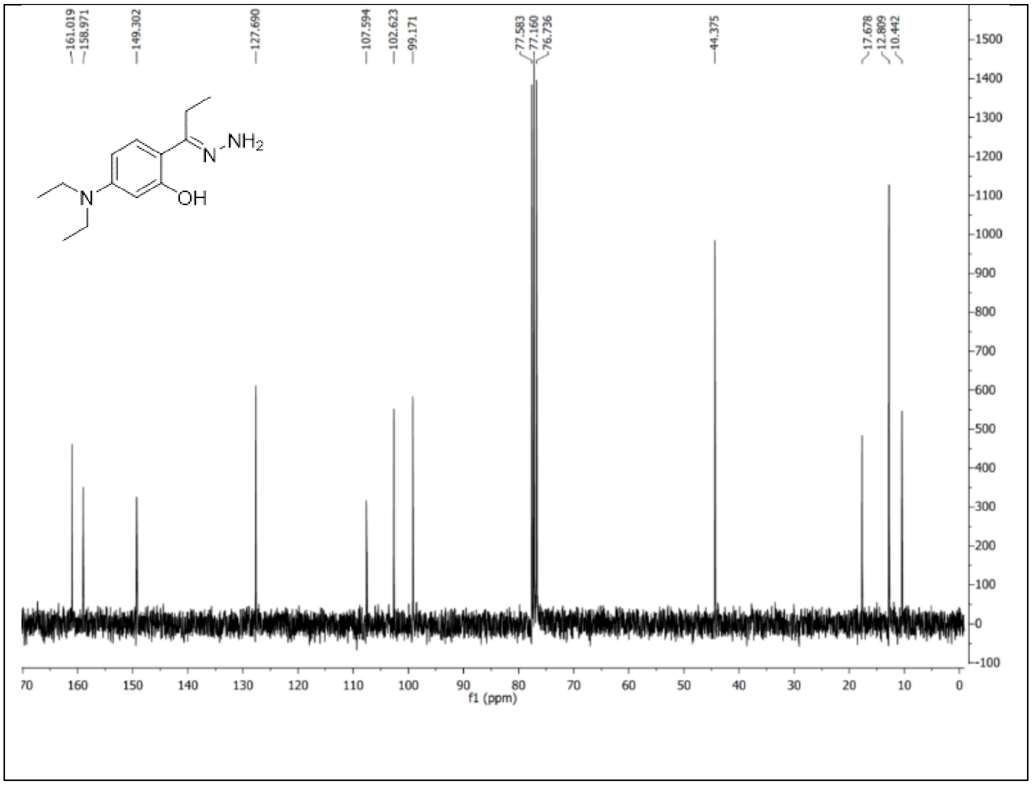

Supplementary Figure S25. ^{13}C-NMR of compound **5** (75 MHz, $CDCl_3$).

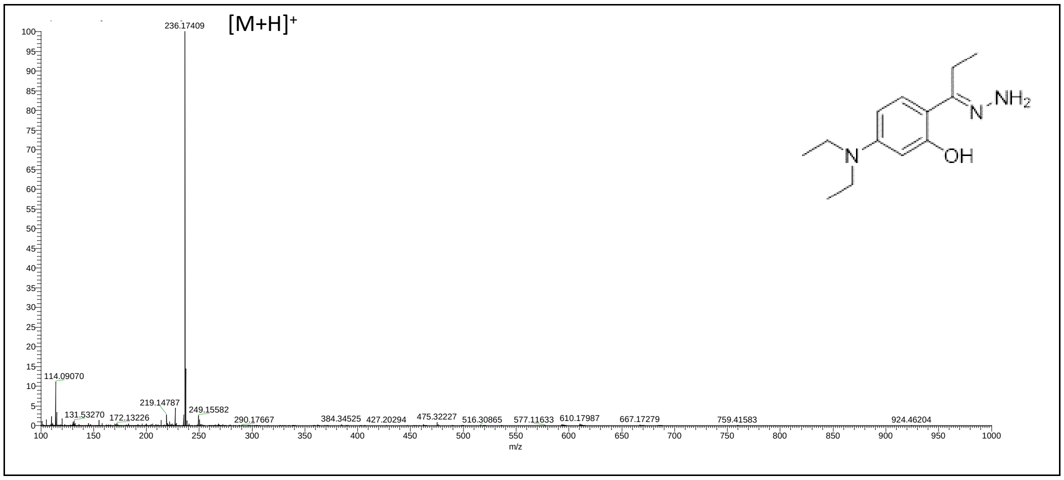

Supplementary Figure S26. High Resolution Mass Spectrum of compound **5**.

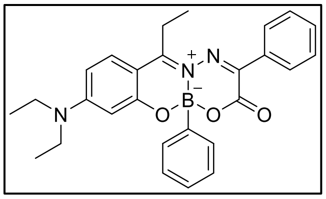

BASHY 2. Orange solid; Yield 82 %; **m.p.** 213-214 ºC; **^{1}H NMR** (300 MHz, $CDCl_3$) δ 8.05 – 7.97 (m, 2H, $CH_{aromatic}$), 7.56 – 7.28 (m, 6H, $CH_{aromatic}$), 7.23 – 7.12 (m, 3H, $CH_{aromatic}$), 6.36 (dd, *J* = 10, 3 Hz, 1H, $CH_{aromatic}$), 6.20 (d, *J* = 3 Hz, 1H, $CH_{aromatic}$), 3.53 – 3.33 (m, 5H, -CC$\underline{H}_2$CH$_3$ and -N(C$\underline{H}_2$)$_2$(CH$_3$)$_3$), 3.23 – 3.10 (m, 1H, -CC$\underline{H}_2$CH$_3$), 1.38 (t, *J* = 8 Hz, 3H, -CCH$_2$C$\underline{H}_3$), 1.22 (t, *J* = 7 Hz, 6H, -N(CH$_2$)$_2$(C$\underline{H}_3$)$_3$); **^{13}C NMR** (75 MHz, $CDCl_3$) δ 169.4, 161.3, 156.2, 156, 152.7, 133.2, 131.5, 130.9, 130.8, 129.5, 128.2, 127.6, 127.6, 107.3, 105.5, 99.2, 45.1, 20.2, 13.2, 12.8; **HRMS** calcd m/z ([M+Na]$^+$): 476.2116, found m/z ([M+Na]$^+$): 476.2104.

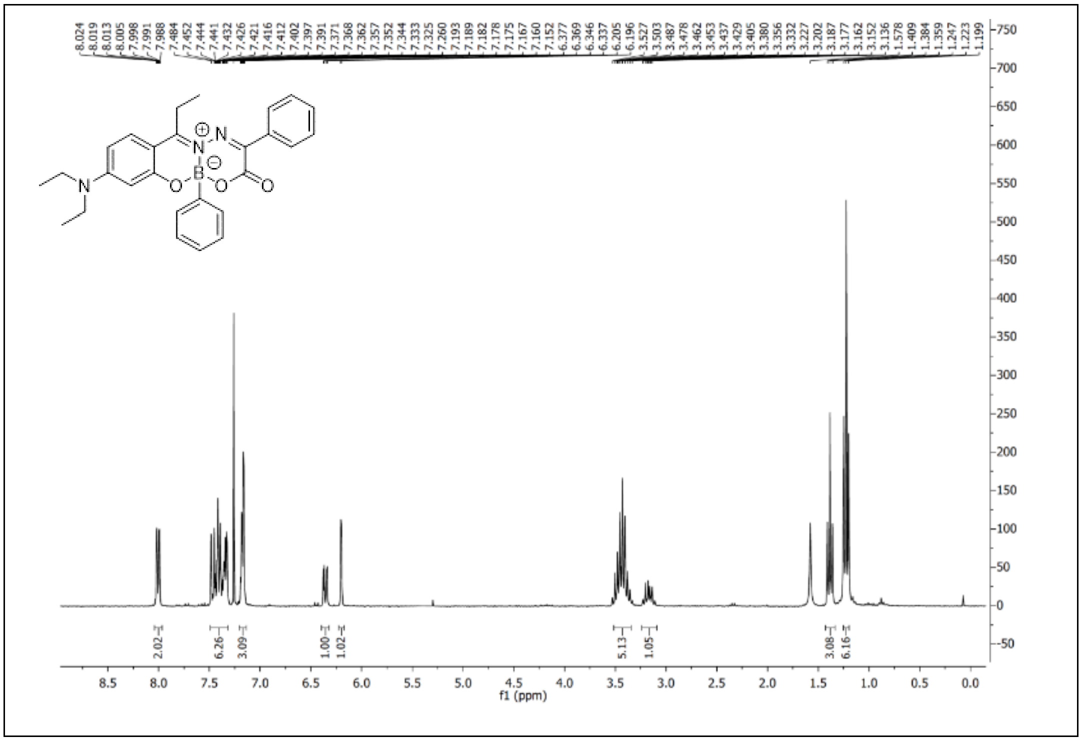

Supplementary Figure S27. ^{1}H-NMR of BASHY td**2** (300 MHz, $CDCl_3$).

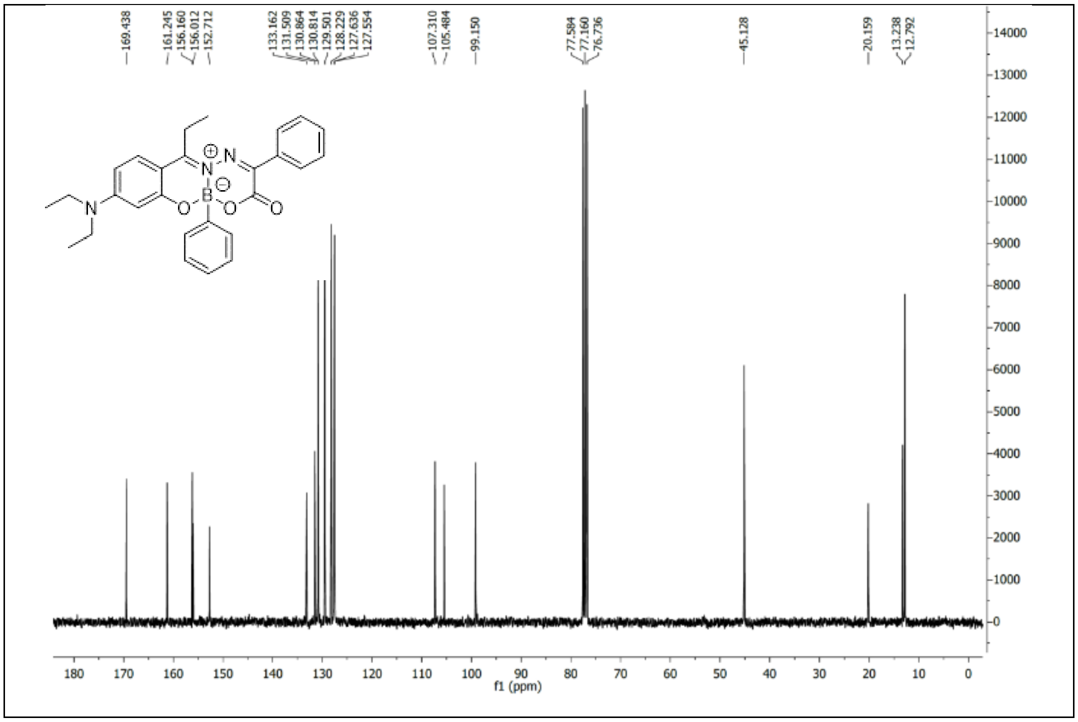

Supplementary Figure S28. ^{13}C-NMR of BASHY td**2** (75 MHz, $CDCl_3$).

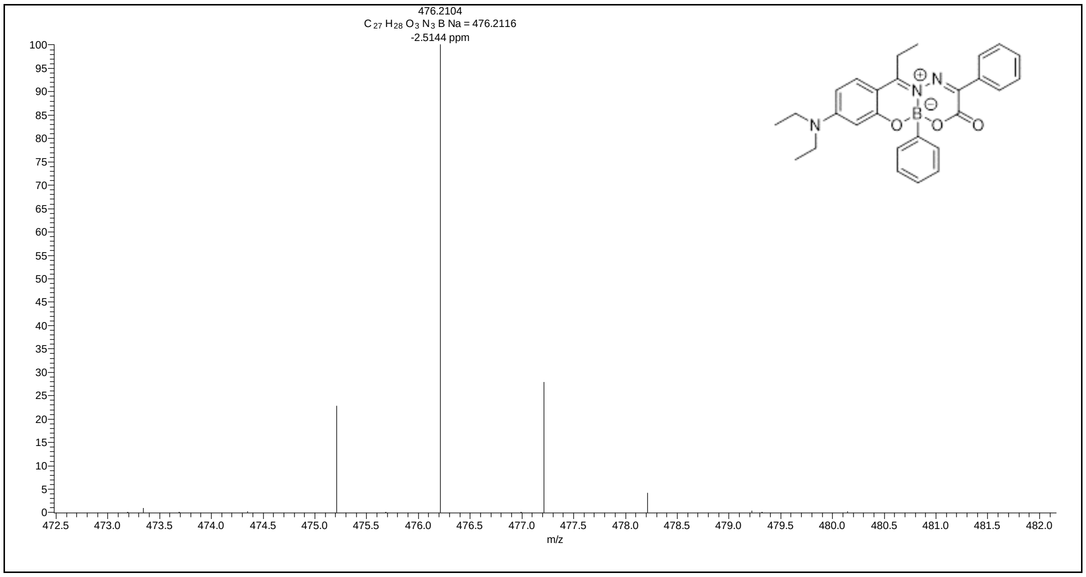

Supplementary Figure S29. High Resolution Mass Spectrum of BASHY td**2**.

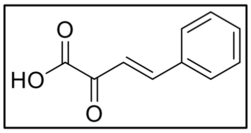

Compound 6 – Yellow solid; Yield 73%; **m.p.** 61-62 ºC; ^{1}**H NMR** (300 MHz, $CDCl_3$) δ 8.13 (d, *J* = 16 Hz, 1H, CH_{vinyl}), 7.74 – 7.65 (m, 2H, $CH_{aromatic}$), 7.60 (d, *J* = 16 Hz, 1H, CH_{vinyl}), 7.55 – 7.40 (m, 3H, $CH_{aromatic}$); **LRMS** calcd m/z ([M-H]$^-$): 175, found m/z ([M-H]$^-$): 175.

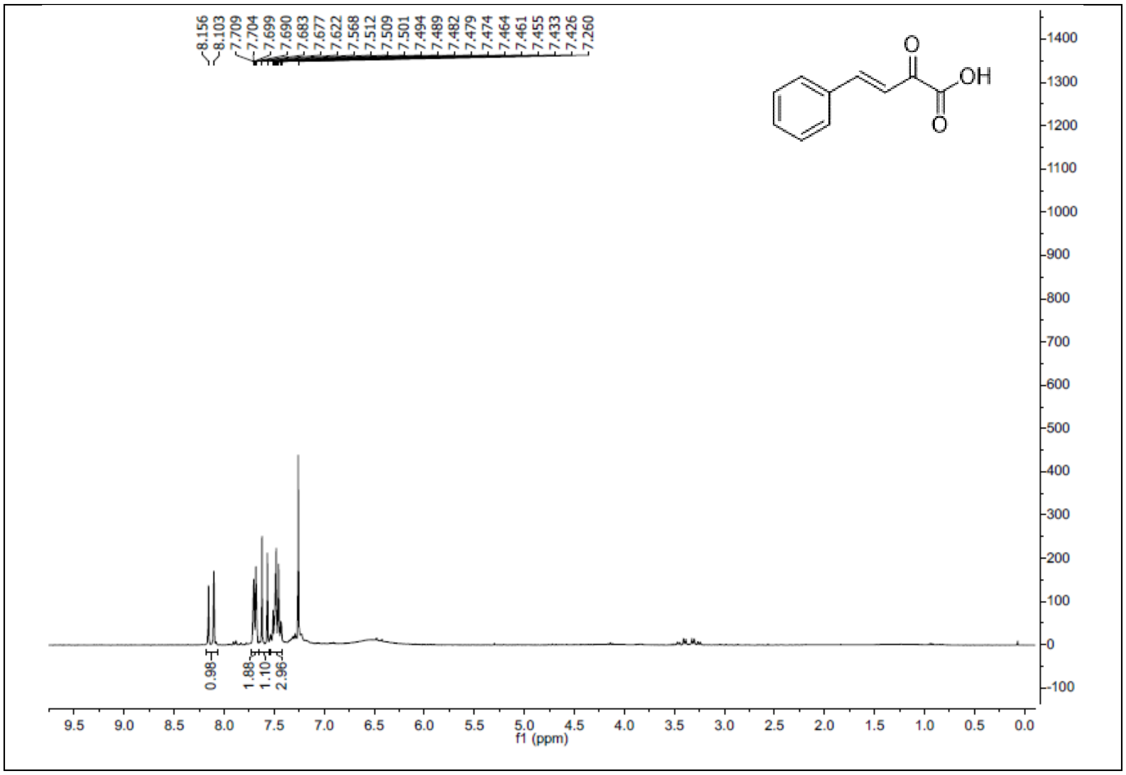

Supplementary Figure S30. ^{1}H-NMR of compound **6** (300 MHz, $CDCl_3$).

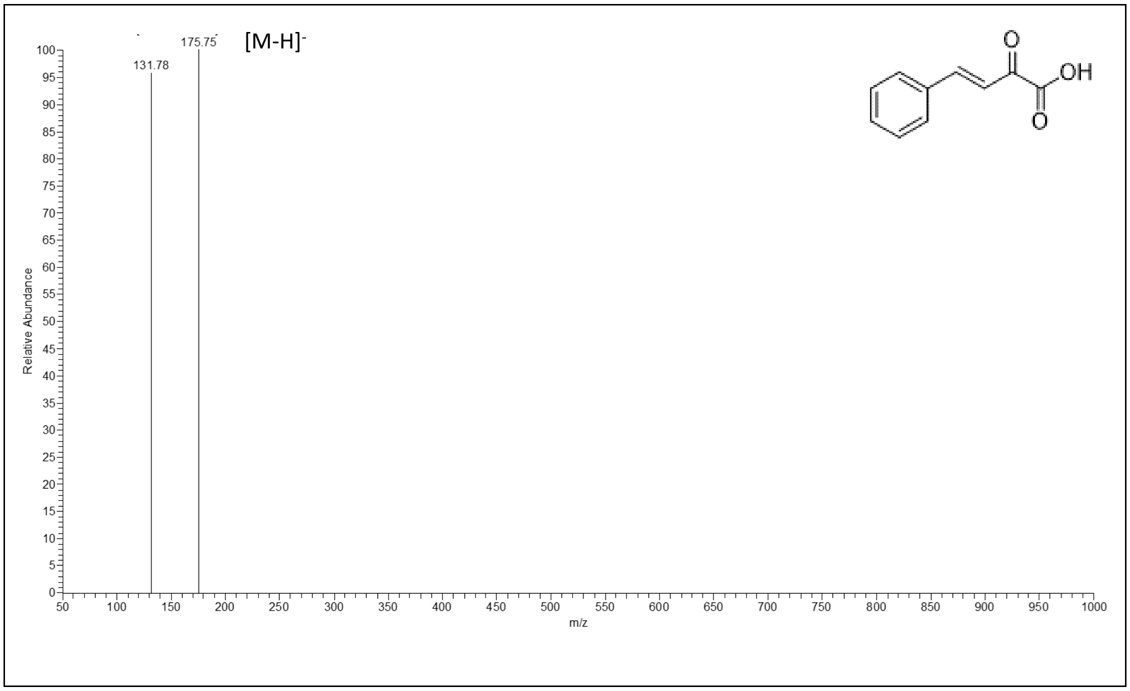

Supplementary Figure S31. Low Resolution Mass Spectrum of compound **6**.

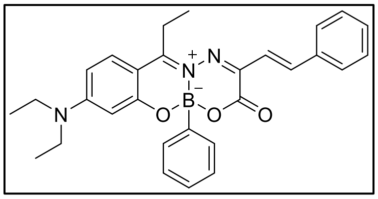

BASHY 3. Red solid; Yield 71 %; **m.p.** 189-190 ºC; **^{1}H NMR** (300 MHz, $CDCl_3$) δ 7.81 (d, *J* = 17 Hz, 1H, CH_{vinyl}), 7.59 – 7.53 (m, 2H, $CH_{aromatic}$), 7.45 (d, *J* = 9 Hz, 1H, $CH_{aromatic}$), 7.40 – 7.28 (m, 5H, $CH_{aromatic}$), 7.22 – 7.15 (m, 4H, $CH_{aromatic+vinyl}$), 6.35 (dd, *J* = 9, 3 Hz, 1H, $CH_{aromatic}$), 6.19 (d, *J* = 3 Hz, 1H, CH_{arom}), 3.51 – 3.34 (m, 5H, $-CC\underline{H}_2CH_3$ and $-N(C\underline{H}_2)_2(CH_3)_3$), 3.22 – 3.08 (m, 1H, $-CC\underline{H}_2CH_3$), 1.36 (t, *J* = 8 Hz, 3H, $-CCH_2C\underline{H}_3$), 1.28 – 1.15 (m, 6H, $-N(CH_2)_2(C\underline{H}_3)_3$); **^{13}C NMR** (75 MHz, $CDCl_3$) δ 168.9, 161.2, 156.6, 156, 150.9, 139.2, 136.2, 131.4, 130.9, 129.6, 128.9, 127.9, 127.7, 127.6, 121.3, 107.2, 105.8, 99.3, 77.6, 77.2, 76.7, 45.2, 20.1, 13.3, 12.9; **HRMS** calcd m/z ($[M+Na]^+$): 502.2272, found m/z ($[M+Na]^+$): 502.2260.

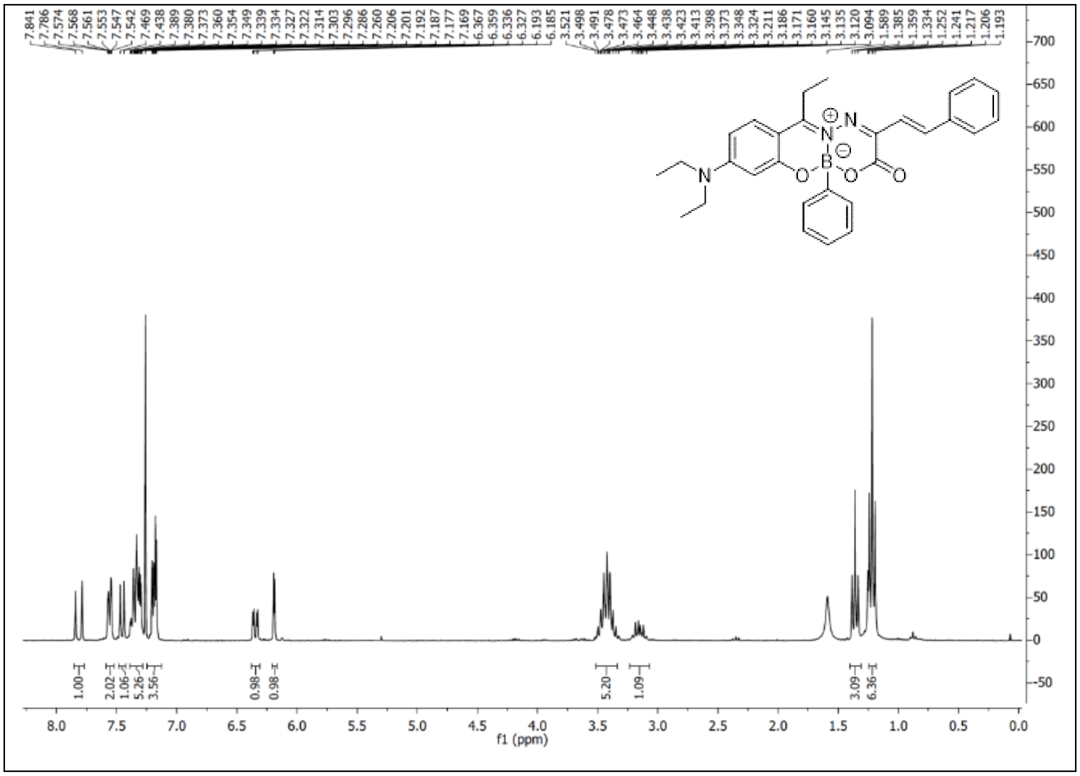

Supplementary Figure S32. ^{1}H-NMR of BASHY td**3** (300 MHz, $CDCl_3$).

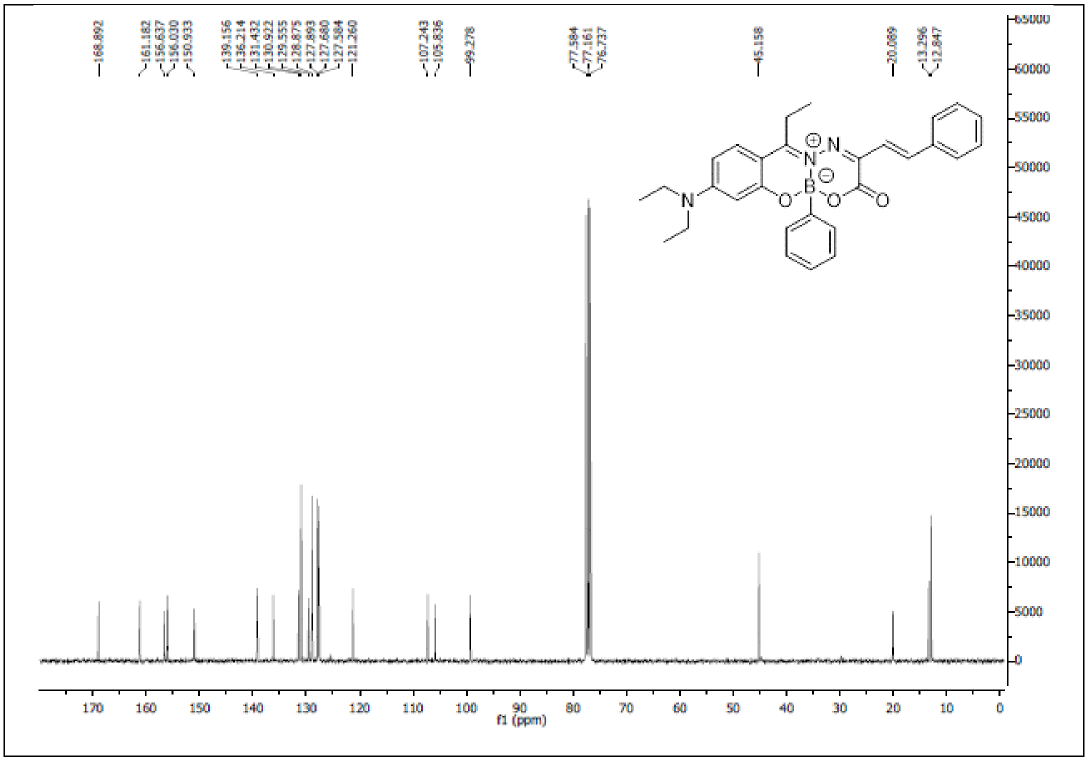

Supplementary Figure S33. ^{13}C-NMR of BASHY td**3** (75 MHz, $CDCl_3$).

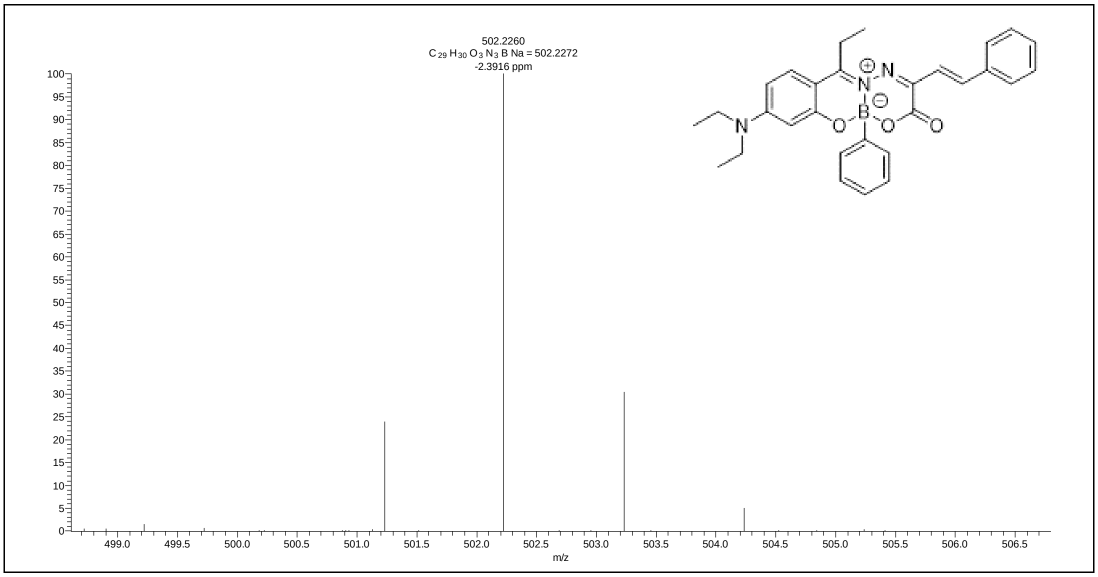

Supplementary Figure S34. High Resolution Mass Spectrum of BASHY td**3**.

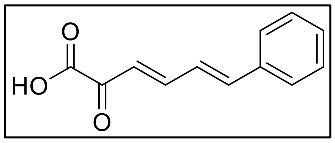

Compound 7. Orange solid; Yield 77%; **m.p.** 77-78 ºC; **^{1}H NMR** (300 MHz, $CDCl_3$) δ 7.95 (dd, *J* = 15, 11 Hz, 1H, CH_{vinyl}), 7.57 – 7.48 (m, 2H, $CH_{aromatic}$), 7.44 – 7.35 (m, 3H, $CH_{aromatic}$), 7.24 – 6.97 (m, 3H, CH_{vinyl}); **LRMS** calcd m/z ([M+H]$^+$): 203, found m/z ([M+H]$^+$): 203.

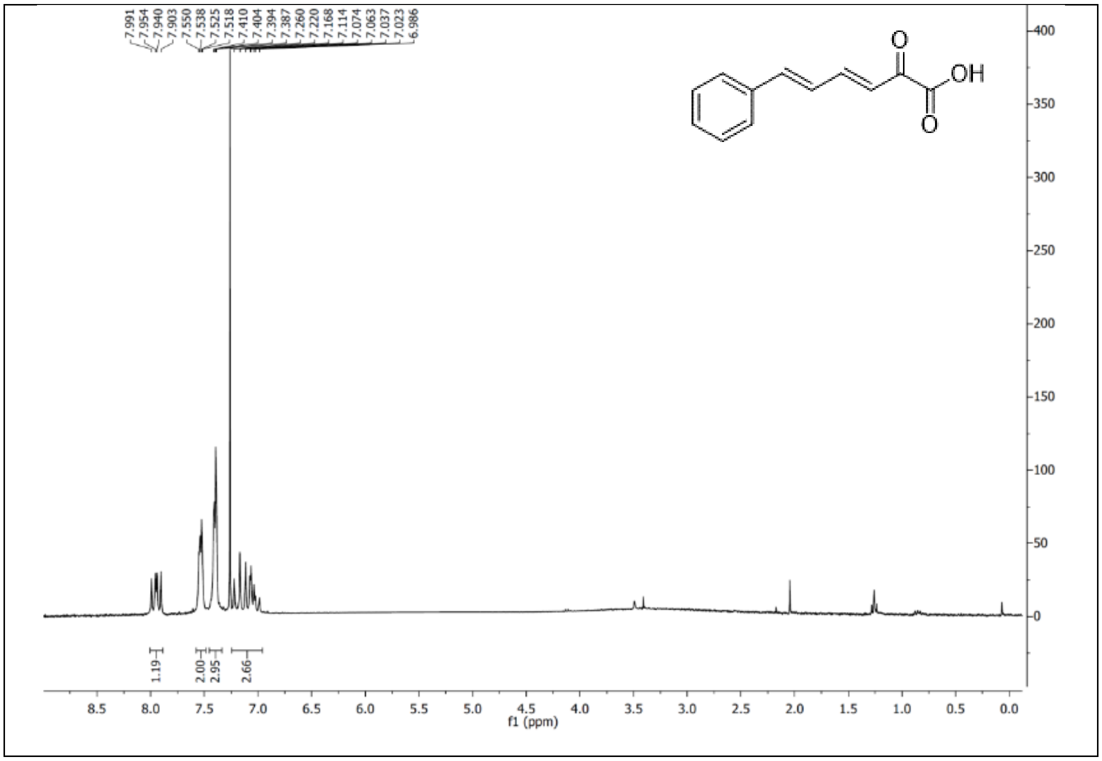

Supplementary Figure S35. ^{1}H-NMR of compound **7** (300 MHz, $CDCl_3$).

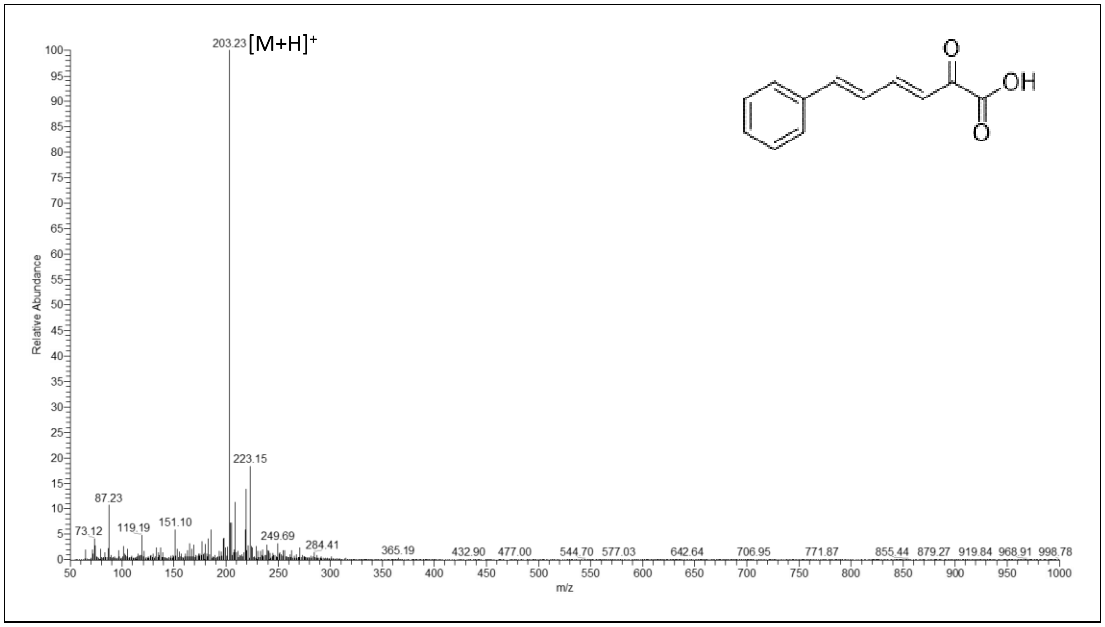

Supplementary Figure S36. Low Resolution Mass Spectrum of compound **7**.

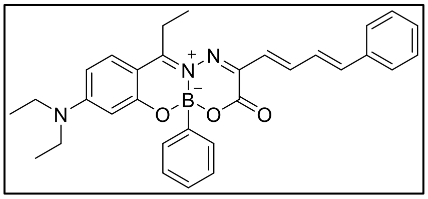

BASHY 4. Dark red solid; Yield 70 %; **m.p.** 206-207 ºC; **^{1}H NMR** (300 MHz, $CDCl_3$) δ 7.63 (dd, *J* = 16, 10 Hz, 1H, CH_{vinyl}), 7.48 – 7.41 (m, 3H, $CH_{aromatic}$), 7.38 – 7.27 (m, 5H, $CH_{aromatic}$), 7.21 – 7.15 (m, 3H, $CH_{aromatic}$), 7.01 – 6.74 (m, 3H, CH_{vinyl}), 6.34 (dd, *J* = 9, 3 Hz, 1H, $CH_{aromatic}$), 6.18 (d, *J* = 3 Hz, 1H, $CH_{aromatic}$), 3.51 – 3.32 (m, 5H, -$C\underline{H}_2CH_3$ and -$N(C\underline{H}_2)_2(CH_3)_3)$), 3.21 – 3.06 (m, 1H, -$CC\underline{H}_2CH_3$), 1.35 (t, *J* = 8 Hz, 3H, -$CCH_2C\underline{H}_3$), 1.29 – 1.16 (m, 6H, -$N(CH_2)_2(C\underline{H}_3)_3)$); **^{13}C NMR** (75 MHz, $CDCl_3$) δ 168.6, 161.1, 156.6, 156, 150.8, 139.8, 138.6, 136.7, 131.4, 130.9, 128.9, 128.8, 128.7, 127.7, 127.6, 127.1, 125.3, 107.2, 105.9, 99.3, 45.1, 20, 13.2, 12.8; **HRMS** calcd m/z ($[M+Na]^+$): 528.2429, found m/z ($[M+Na]^+$): 528.2418.

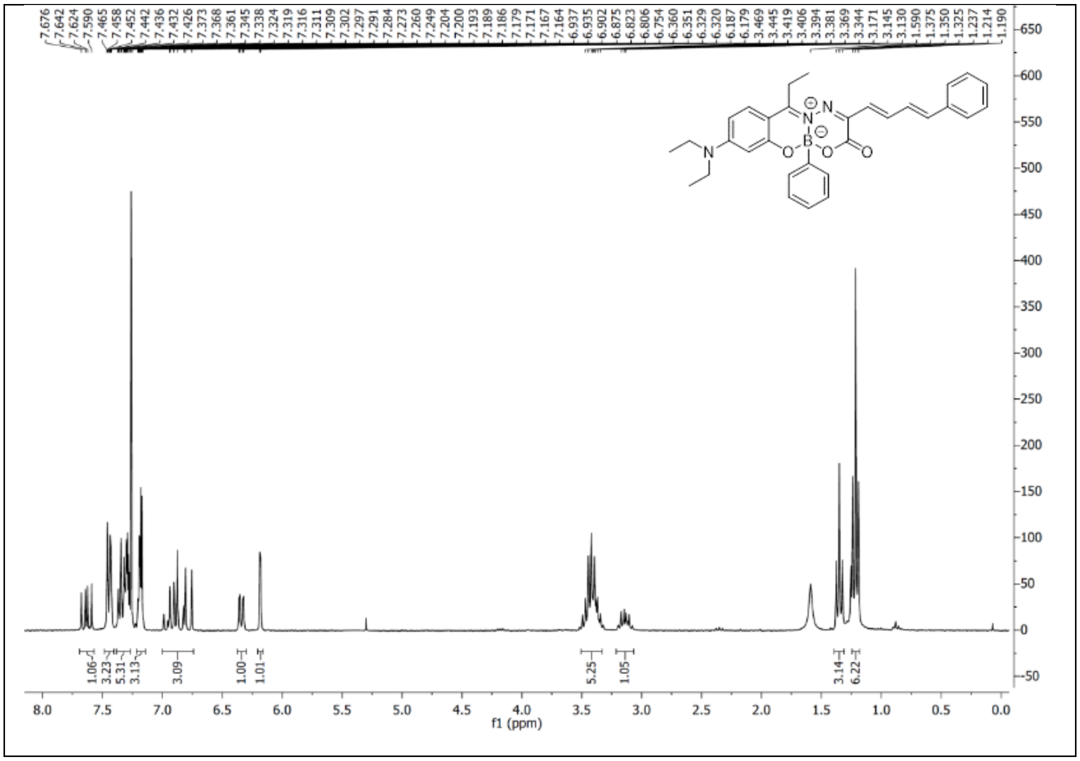

Supplementary Figure S37. ^{1}H-NMR of BASHY td**4** (300 MHz, $CDCl_3$).

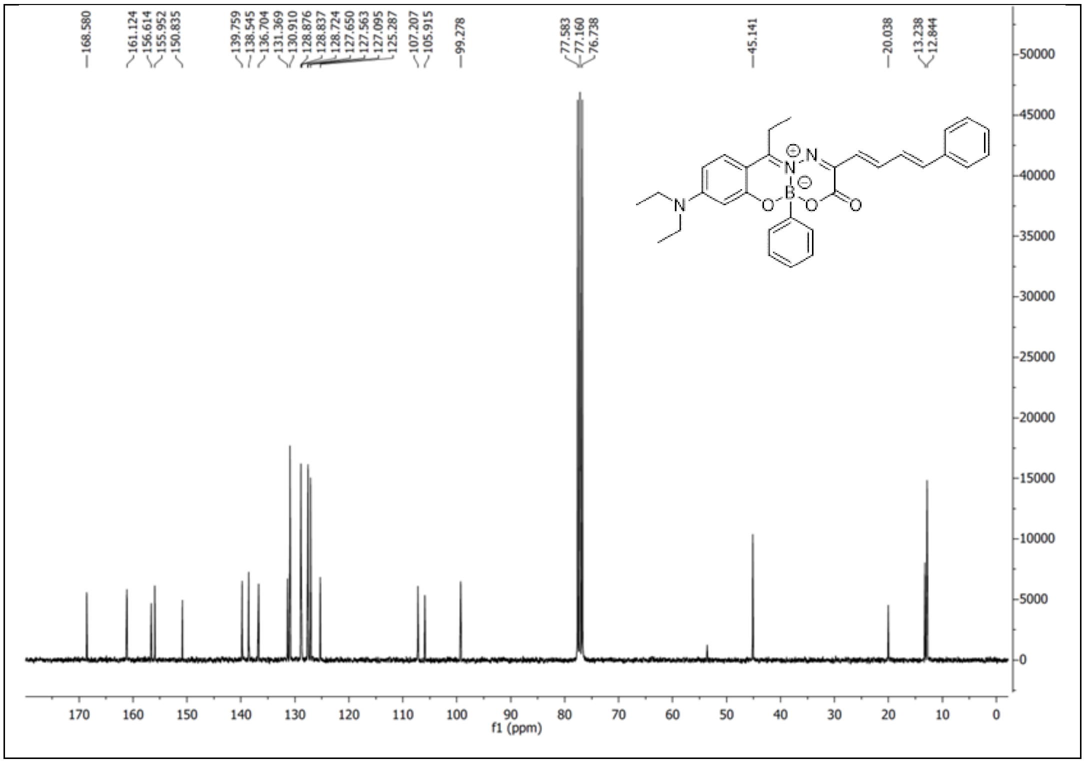

Supplementary Figure S38. ^{13}C-NMR of BASHY td**4** (75 MHz, $CDCl_3$).

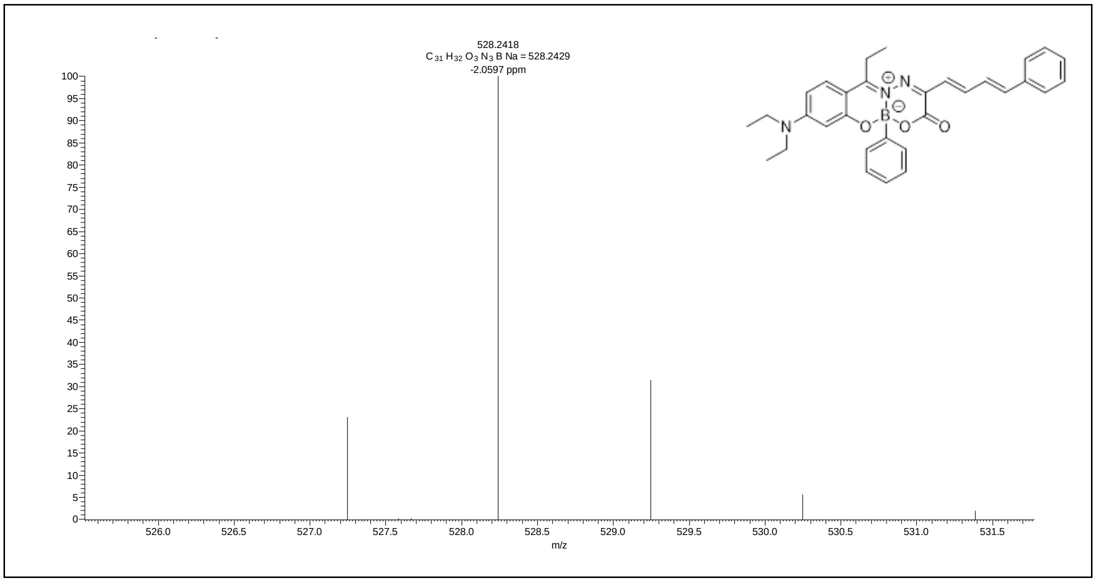

Supplementary Figure S39. High Resolution Mass Spectrum of BASHY td**4**.

Structural characterization of BASHY dyes

Supplementary Table

Supplementary Table S1. Selected photophysical data of the td**1-4** in apolar medium (toluene).

	$\lambda_{max,abs}$ (nm) [a]	$\lambda_{max,f}$ (nm) [b]	Φ_f [c]	brightness $\varepsilon \times \Phi_f$ ($M^{-1}cm^{-1}$)	contrast [d] $\Phi_{f,apolar}/\Phi_{f,polar}$
Td**1** [e]	471	508	0.60	37000	>60
Td**2** [f]	472	509	0.72	35000	24
Td**3** [f]	497	531	0.58	34000	12
Td**4** [f]	513	548	0.32	19000	5

[a] UV/vis absorption maximum. [b] Fluorescence emission maximum. [c] Fluorescence quantum yield. [d] Quotient between the fluorescence quantum yield in apolar medium (toluene) and polar medium (methanol). [e] Data from [19]. [f] Data from [30]. The fluorescence quantum yields in methanol (0.03 for td**2**, 0.05 for td**3**, and 0.06 for td**4**), used for the determination of the “contrast” parameter, were measured for this work following the same method as described in [30].
